# Exercise intervention improves fertility outcomes in females and males: an umbrella review of meta-analyses

**DOI:** 10.7189/jogh-16-04282

**Published:** 2026-08-28

**Authors:** Lai Kwan Lam, Bekalu Kassie Alemu, Margie H Davenport, Chi Chiu Wang, Yiu Leung Chan, Yao Wang

**Affiliations:** 1 Department of Obstetrics and Gynaecology, Faculty of Medicine, The Chinese University of Hong Kong, Hong Kong SAR, China; 2 Program for Pregnancy and Postpartum Health, Faculty of Kinesiology, Sport, and Recreation, University of Alberta, Edmonton, Canada; 3 Li Ka Shing Institute of Health Sciences, Faculty of Medicine, The Chinese University of Hong Kong, Hong Kong SAR, China; 4 Assisted Reproductive Technology Unit, Department of Obstetrics and Gynaecology, Faculty of Medicine, The Chinese University of Hong Kong, Hong Kong SAR, China

**Keywords:** exercise, physical activity, fertility, reproductive health, sperm quality, umbrella review

## Abstract

**Background:**

Infertility is a major global health burden, yet scalable lifestyle strategies to improve reproductive outcomes remain limited. Exercise is low cost and biologically plausible, but evidence on its association with fertility-related outcomes is dispersed across multiple meta-analyses. We therefore conducted an umbrella review of meta-analyses of randomised controlled trials evaluating exercise and fertility-related outcomes in females and males.

**Methods:**

Six databases (PubMed, Cochrane Library, Web of Science, Scopus, EBSCOhost, and Google Scholar) were searched from database inception to 24 November 2025. Eligible studies were systematic reviews with meta-analyses of randomised controlled trials. Overlap among reviews was managed using citation matrices. Methodological quality, risk of bias, and certainty of evidence were assessed using AMSTAR 2, ROBIS, and GRADE, respectively.

**Results:**

Thirteen meta-analyses were included, covering 24,470 females and 8068 males. In females, exercise showed positive effects in reproductive hormonal profiles, including sex hormone-binding globulin and anti-Müllerian hormone. It also associated with higher ovulation rate, pregnancy rate and clinical pregnancy rate. In males, exercise was linked to amelioration in sperm quality, regarding concentration, morphology, count, and semen volume. Moreover, exercise in male participants was accompanied by higher pregnancy rate in their female partners.

**Conclusions:**

Exercise is associated with improved fertility and reproductive health outcomes in both females and males, supporting its potential role as an adjunctive, low-cost, noninvasive component of fertility management. However, the certainty of evidence ranged from high to very low across outcomes, with most evidence rated as low or very low certainty. Heterogeneity across populations and exercise intervention components also limited confidence in these findings. Further high-quality trials are needed to clarify optimal exercise prescriptions and strengthen the certainty of evidence.

**Registration:**

PROSPERO: CRD42025638675

Infertility is defined as the inability to conceive after 12 months of regular unprotected intercourse [1], affecting approximately 48.5 million couples worldwide [2]. The prevalence of infertility has risen in recent decades, from 3.5% to 16.7% in developed regions and from 6.9% to 9.3% in developing countries [3]. Accumulating evidence indicates that infertility is not merely a reproductive issue but is also associated with elevated psychological distress, including stress, anxiety, and depression [4], further affecting overall health. As a growing global concern, infertility has been recognised within the World Health Organization’s reproductive health agenda and the United Nations Sustainable Development Goals [5,6]. Although *in vitro* fertilisation (IVF) is widely used to support conception [7], its high financial cost and limited accessibility underscore the need for safe, scalable, and noninvasive adjunctive strategies to improve fertility capacity.

Exercise refers to structured physical activity (PA) intended to enhance or maintain overall health [8]. It confers metabolic benefits by improving cardiorespiratory function, reducing oxidative stress, and alleviating systemic inflammation [9]. However, the relationship between exercise and fertility capacity remains unclear. Although previous studies have reported that exercise improves ovulation [10] in women with polycystic ovary syndrome (PCOS) and enhances endometrial receptivity in healthy females [11–13], direct evidence linking exercise to key fertility outcomes, such as pregnancy rate, remains limited. In males, moderate-intensity exercise has been associated with improved sperm motility and concentration [14,15], whereas other studies have suggested that exercise-induced thermal stress and chronic hypoxia may impair sperm DNA integrity [16–18]. Moreover, prior umbrella reviews have evaluated the effects of physical activity on maternal health and male infertility treatment [19–21], but the influence of exercise on reproductive outcomes, including ovulation rates, conception rates, hormonal profiles, and semen quality, remains largely unexplored. Therefore, an updated umbrella review is needed to systematically summarise and appraise the existing evidence on exercise and fertility-related outcomes in both females and males.

In this umbrella review, we synthesised latest evidence from systematic reviews with meta-analyses of randomised controlled trials (RCTs) on exercise interventions for reproductive health and fertility outcomes in both males and females. Outcomes of interest included ovulation, pregnancy-related outcomes, reproductive hormonal profiles, semen parameters, and partner pregnancy outcomes. We further examined the effects of training modality and intensity on reproductive health.

## METHODS

The study protocol was preregistered with the International Prospective Register of Systematic Reviews (PROSPERO: CRD42025638675). This review was conducted in accordance with the PRISMA statement.

### Search strategy

A comprehensive systematic search was conducted to identify systematic reviews and meta-analyses of RCTs evaluating the effects of exercise-based interventions on fertility outcomes in females and males. Searches were performed across six electronic databases: PubMed, Scopus, EBSCO, Cochrane Library, Web of Science, and Google Scholar. Searches covered all available publications from database inception to 24 November 2025, used the same core search terms, and screened records in order of relevance until no further eligible records were identified. The search was supplemented by manual screening of the reference lists of all included systematic reviews and meta-analyses, as well as relevant review articles identified during full-text assessment, to identify potentially eligible additional reviews. Any additional records identified through hand-searching were documented and included in the flow diagram. Search terms included combinations of keywords such as ‘exercise’, ‘physical activity’, ‘fertility’, ‘reproductive health’, and ‘infertility’. Boolean operators (AND, OR) were used to refine the search, and Medical Subject Headings (MeSH) terms were applied where applicable (Table S1 in the **Online Supplementary Document**). The retrieved records from each database were transferred to EndNote 21 software, where duplicates were removed and records were managed before screening. Two reviewers (LKL and BKA) independently screened the records by title, abstract, and full text based on the pre-specified eligibility criteria. Any disagreements during screening were resolved through discussion or consultation with a third reviewer (YW). Findings were reported in accordance with the Preferred Reporting Items for Systematic Reviews and Meta-Analyses (PRISMA) guidelines [22].

### Eligibility criteria

This study followed the population, intervention, comparison, outcomes, and study design (PICOS) framework.

### Population

The population of interest included reproductive-aged females and males with fertility challenges. Reproductive age was defined as 18 to 49 years for females and 18 years or older for males, unless otherwise defined by the included reviews. Fertility challenges were defined as infertility diagnosed according to standard clinical criteria, subfertility, impaired reproductive function, or fertility-related conditions such as polycystic ovary syndrome (PCOS), obesity-related infertility, abnormal semen parameters, or fertility treatment among couples.

### Intervention (exposure)

The interventions analysed in this review encompassed all prescribed forms of exercise, ranging from structured physical activity programmes to interventions combining exercise or physical activity with guided regimens. This inclusive approach ensured a comprehensive evaluation of the role of exercise in influencing reproductive outcomes. Studies in which exercise was a component of broader interventions, such as those combined with dietary changes, pharmacological treatments, or alternative therapies, were eligible for inclusion, provided that exercise-related or combined effects were assessed or reported. Non-exercise-based interventions, such as those exclusively involving dietary changes, pharmacological treatments, or alternative therapies, were excluded. For the control group, this review considered studies with clearly defined comparison conditions that differed from the intervention group, such as no intervention, usual care, alternative non-exercise interventions, combined interventions without a structured exercise component, or specific clinical procedures.

### Comparison

Eligible comparators included no intervention, usual care, wait-list control, placebo or minimal intervention, and active non-exercise comparators. For clarity, comparator conditions were described in two broad categories:

• inactive or usual-care comparators, including no intervention, usual care, wait-list control, placebo, or minimal advice;

• and active comparators, including dietary, pharmacological or medical, behavioural, complementary, clinical, or other non-exercise interventions, as well as control groups receiving the same background treatment without the exercise component.

Where applicable, comparisons involving different exercise or physical activity interventions were considered active comparisons rather than inactive or usual-care controls. This distinction was made to reduce clinical heterogeneity and avoid attributing the effects of co-interventions solely to exercise.

### Outcomes

Eligibility criteria were defined using the population, intervention, comparison, outcomes, and study design (PICOS) framework. The population comprised reproductive-aged women and men with fertility concerns. The intervention included prescribed exercise or physical activity programmes; multicomponent interventions (*e.g.* exercise plus dietary counselling) were eligible if exercise-related effects were reported, whereas non-exercise-only interventions (*e.g.* diet alone or pharmacotherapy alone) were excluded. Comparators included no intervention, usual care, or alternative non-exercise interventions. Outcomes covered female reproductive outcomes (*e.g.* ovulation, implantation, clinical pregnancy, ongoing pregnancy), male reproductive outcomes (*e.g.* sperm concentration, progressive motility, normal morphology, DNA fragmentation index), and endocrine profiles (*e.g.* follicle-stimulating hormone, luteinizing hormone, testosterone, oestradiol, anti-Müllerian hormone). Studies that did not report any of these outcomes were excluded.

### Study design

Systematic reviews and meta-analyses of randomised controlled trials reporting pairwise meta-analyses were eligible for inclusion. Reviews that additionally reported meta-regression or network meta-analysis were also eligible. Reviews without fertility-related outcomes or unavailable full texts were excluded. Only articles published in English or Chinese were considered.

### Study selection

Duplicates were removed before screening. Two reviewers (LKL and BKA) independently examined the titles and abstracts of all retrieved studies using a standardised protocol. Studies considered eligible by at least one reviewer proceeded to full-text review. The full-text articles were then independently reviewed by both reviewers to determine eligibility. Any disagreements about study inclusion were resolved through discussion between the reviewers. If consensus could not be reached, a third reviewer (YW) was consulted to make the final decision. This systematic process ensured that all selected studies adhered to the predefined eligibility criteria.

### Data extraction

Two reviewers (LKL and BKA) independently performed data extraction using a standardised protocol based on a predefined extraction form developed for this umbrella review. The extraction form included predefined variables and coding categories to ensure consistency across reviewers. Extracted information included study characteristics, population characteristics, intervention details, comparator categories, outcomes, effect estimates, heterogeneity measures, and publication bias assessments. Discrepancies in extracted data or interpretation were resolved through structured consensus discussions, with unresolved cases escalated to a third reviewer (YW). The extraction framework captured four primary domains: study characteristics, population, intervention, and outcomes. Study characteristics included author names, publication year, demographic profiles of target populations, participant or study sample sizes, and intervention specifics such as exercise modality, session frequency, and programme duration. The population domain focused on the demographic and clinical profiles of the target populations. The intervention domain described the general structure and components of the exercise programme. Clinical fertility outcomes were analysed to evaluate the interventions, ensuring alignment with PICO principles. Analytical parameters included quantitative measures of heterogeneity (*I*^2^ statistic), effect size estimation models (fixed or random effects), and publication bias assessments using Egger's and Begg's statistical tests.

For this umbrella review, exercise interventions were defined as planned, structured, and repetitive physical activities prescribed to improve fitness, health, or fertility-related outcomes. General physical activity or lifestyle counselling was not considered exercise unless a structured exercise prescription was reported. Exercise training modes were classified according to the American College of Sports Medicine (ACSM) [23] as cardiorespiratory (aerobic) exercise (*e.g.* walking, cycling), resistance training (RT, *e.g.* weights, bands, body weight), flexibility training (stretching), and neuromotor training (*e.g.* yoga, Pilates). Where intensity was specified, aerobic protocols were further categorised as moderate-intensity continuous training (MICT), high-intensity continuous training (HICT), or high-intensity interval training (HIIT). Multimodal programmes were coded as combined training, such as combined aerobic and resistance training (CET).

### Overlap management

We performed overlap analysis to address redundancy using the Five-Step Framework [24]. A citation matrix was constructed to visualise overlapping studies across the included systematic reviews and meta-analyses. Rows in the citation matrix represented unique primary studies, and columns represented the included systematic reviews and meta-analyses. A coloured cell indicated that a primary study was included in the corresponding review. The corrected covered area (CCA) was calculated to quantify the extent of overlap. CCA was calculated as (N − r) divided by (r × c − r), multiplied by 100, where N represents the total number of primary study occurrences across reviews, r represents the number of unique primary studies, and c represents the number of included reviews. CCA scores were classified as slight (CCA = 0–5%), moderate (CCA = 6–10%), high (CCA = 11–15%), or very high (CCA>15%). If high or very high overlap was encountered, we selected the most recent meta-analysis as the primary source to maximise the breadth and depth of evidence with limited redundancy. When overlap was slight, all eligible reviews were retained, and differences in definitions, scope, and methods were considered during narrative interpretation. When CCA alone was insufficient to determine exclusion, two independent reviewers conducted a detailed full-text review of the overlapping meta-analyses.

### Quality and strength of evidence assessment

To assess the methodological quality, risk of bias, and certainty of evidence of the included review-level evidence, three validated tools were used. A Measurement Tool to Assess Systematic Reviews 2 (AMSTAR 2) [25] was used to evaluate the methodological quality and rigor of the included systematic reviews and meta-analyses. Risk of Bias in Systematic Reviews (ROBIS) [26] was applied only at the review level to assess the risk of bias in the included systematic reviews and meta-analyses, not in the individual primary studies contained within those reviews. This tool assesses concerns across four domains: study eligibility criteria, identification and selection of studies, data collection and study appraisal, and synthesis and findings, followed by an overall judgment of risk of bias. Overall ROBIS judgments were categorised as low, high, or unclear risk of bias according to ROBIS guidance. Grading of Recommendations, Assessment, Development, and Evaluations (GRADE) [27] was used to appraise the certainty of evidence for each outcome. The ratings from ROBIS were not used as exclusion criteria; instead, they informed the narrative synthesis and interpretation of the robustness of the evidence, with findings from reviews judged to be at high or unclear risk of bias interpreted with greater caution. Any discrepancies in quality assessment were resolved through discussion or, when necessary, consultation with a third reviewer (YW).

### Diagram construction

To investigate the relationships between exercise modalities and reproductive health outcomes, we constructed an alluvial diagram using OriginPro 2025 to visually represent the connections and relative contributions of different exercise types to various reproductive health parameters. Nodes in the diagram represented studies, exercise modalities, and reproductive health outcomes. The alluvial diagrams were constructed using positive review-level findings only, and studies were included when sufficient information was available to link the exercise intervention, broader exercise mode, and corresponding reproductive outcome. The thickness of the connecting links was proportional to the magnitude of the effect size or contribution, while colour coding was used to distinguish different studies and exercise modalities, enhancing interpretability. This visualisation approach enabled identification of key patterns, such as the exercise modalities associated with the largest improvements in specific outcomes and overlaps in the effects of different exercise types across studies. By summarising complex relationships between exercise interventions and reproductive health outcomes, the diagram provided an intuitive overview of the data and supported evidence-based recommendations for improving reproductive health.

### Data synthesis

Effect size harmonisation was performed through metric standardisation and variance alignment procedures using *R*, version 4.3.1 (R Foundation for Statistical Computing, Vienna, Austria). Dichotomous outcomes, such as odds ratios (ORs) and risk ratios (RRs), were transformed using natural logarithms to ensure compatibility with continuous outcomes expressed as mean differences (MDs) or standardised mean differences (SMDs). For studies reporting ORs or RRs without explicit variance measures, standard errors were calculated using the Altman-Bland conversion method based on 95% confidence intervals. Where necessary for graphical readability, continuous effect estimates and corresponding 95% confidence intervals were rescaled using the same constant factor. If only one systematic review was available for a specific outcome, the results were reported directly. Heterogeneity for each summary effect estimate was assessed using the *I*^2^ statistic. This heterogeneity was further decomposed into within-cluster heterogeneity, representing effect sizes or multiple arms derived from the same or overlapping reviews, and between-cluster heterogeneity, reflecting variation across unique reviews. *I*^2^ values between 50% and 75% were classified as indicative of moderate heterogeneity, while values exceeding 75% were interpreted as substantial heterogeneity [28]. Additionally, Cochran's Q test (α = 0.05) was used to statistically evaluate the presence of heterogeneity. Narrative sensitivity analyses were conducted to examine the influence of co-interventions and population heterogeneity. Reviews classified as combined interventions were excluded to compare the direction of effects with the primary synthesis. For population-based analysis, only reviews with clearly defined, non-overlapping populations were included.

This study was conducted in adherence to the principles of equity, diversity, and inclusion. Participants in the referenced studies were recruited from diverse backgrounds to ensure representativeness, and the study design aimed to minimise bias and promote inclusivity in all aspects of the research process. Patients and the public were not involved in the design, conduct, reporting, or dissemination plans of this umbrella review because the study synthesised data from previously published studies and did not involve direct interaction with patients or the public.

## RESULTS

### Study selection and characteristics

**Figure 1:**
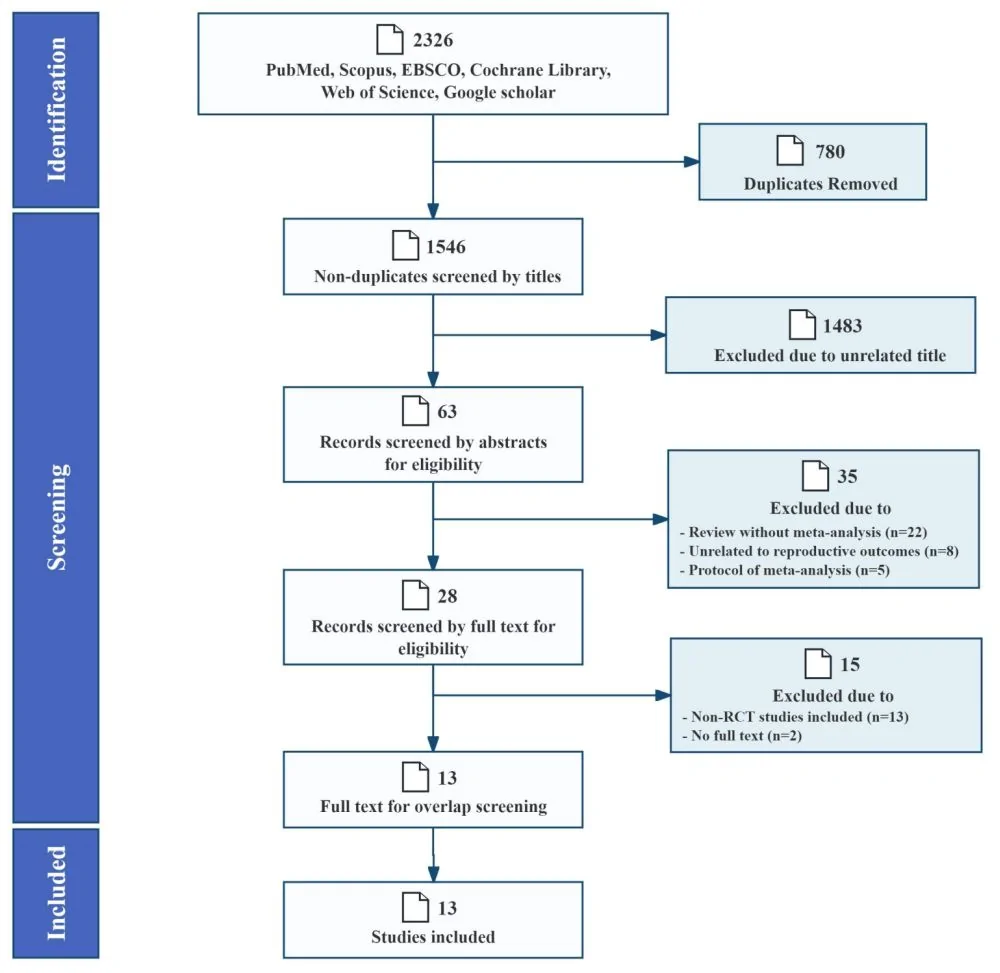
Systematic search and selection of systematic reviews and meta-analyses using a PRISMA flowchart.

The search, screening, and selection process followed the PRISMA flow diagram (**Figure 1**). We retrieved 1546 records from 2326 papers after removing 780 duplicates. A total of 13 systematic reviews and meta-analyses were ultimately included for analysis [29–41], with slight overlap (3.91%) (Table S2 in the **Online Supplementary Document**). All 13 included reviews were based on randomised controlled trials and reported pairwise meta-analyses; among these, 1 also included meta-regression and 2 also included network meta-analyses (**Table 1**). The 13 included reviews involved 32,538 participants, including 24,470 females (75% of the total sample) and 8068 males (25%) (**Table 1**). Among these reviews, 1 was rated as high quality and 12 as moderate quality based on the AMSTAR 2 quality assessment (Table S3 in the **Online Supplementary Document**). At the outcome level, GRADE certainty ranged from high to very low, with most outcomes rated as moderate, low, or very low certainty (Table S4 in the **Online Supplementary Document**). Using ROBIS, 12 included reviews were judged to be at low risk of bias at the review level, whereas 1 review was judged to have an unclear risk of bias. The structured exercise interventions included various exercise modalities (cardiorespiratory, resistance, neuromotor, and combined exercise), intensities (from light to high intensity), and durations ranging from 4 to 52 weeks (**Table 1**).

**Table 1:** Characteristics of meta-analyses of RCTs included in this umbrella review

Author, year	Review type	No. of included studies	Population	No. of sample size (individual)		Exercise intervention	Exercise types	Duration	Intervention period	Collaborative intervention	Outcomes*	ROBIS	AMSTAR scores
				**Women**	**Men**								
Lan 2017 [29]	SRMA of RCTs	8	Obese women	4,559	-	EI/PA	Physical activity, aerobic and resistance training, aerobic training	-	4–24 weeks	DI, PI	1, 2, 3, 4, 5, 6, 7, 8, 9, 10, 11, 12, 13, 14, 33	Low risk	Moderate
Yu 2020 [30]	SRMA of RCTs	8	PCOS-diagnosed women	373	-	EI	Physical activity, structured exercise, high intensity interval training, resistance training	-	-	-	2, 15, 16, 17, 18, 19, 20, 21, 22, 23, 24, 25, 26, 27, 28, 29, 30, 31	Unclear risk	Moderate
Dos Santos 2020 [31]	SRMA of RCTs	10	PCOS-diagnosed women	533	-	EI	Aerobic training, resistance training, high-intensity interval training	90–180 min/week	8–32 weeks	-	1, 2, 15, 17, 26, 27, 28, 29, 30, 32, 33, 34, 35, 36	Low risk	Moderate
Espinós 2020 [32]	SRMA of RCTs	8	Obese infertile women	1,175	-	EI/PA	Structured exercise, moderate-intensity physical activity	-	6–12 weeks	DI, PBS	1, 2, 3, 5, 33, 37, 38, 39, 40	Low risk	High
Hunter 2021 [33]	SRMA of RCTs	15	Obese infertile women	1,253	-	EI	Aerobic training, physical activity, high-intensity interval training, moderate exercise	90–280 min/week	4–48 weeks	DI	1, 3, 4, 5, 33, 37	Low risk	Moderate
Hajizadeh Maleki 2022 [34]	SRMA of RCTs	7	Fertile men and Infertile men	-	2641	EI	Moderate-intensity continuous exercise, high-intensity continuous exercise, high-intensity interval training, resistance exercise, combined aerobic and resistance exercise	45–280 min/week	24 weeks	-	3, 5, 40, 41, 42, 43, 45, 47, 49, 51, 54, 55	Low risk	Moderate
Kazeminia 2022 [35]	SRMA of RCTs	12	PCOS-diagnosed women	577	-	EI/PA	Resistance training, aerobic training, pilates, yoga	120–180 min/week; yoga: 420 min/week	8–24 weeks	-	2, 44	Low risk	Moderate
Chen 2023 [36]	SRMA of RCTs	27	Infertile men	-	4008	EI/PA	Aerobic training, resistance training, combined aerobic and resistance exercise training, high-intensity interval training	-	-	DI, PBA	3, 47, 48, 49, 50, 51	Low risk	Moderate
Sustarsic 2023 [37]	SRMA-MR of RCTs	15	Obese infertile women, obese PCOS-diagnosed women	1,205	-	PA	Aerobic training, resistance training, physical activity	20,000-70,000 steps/weeks; 108–280 min/week	4–24 weeks	DI, PBA	1, 2, 3, 5, 17, 20, 28, 33, 35, 54, 55	Low risk	Moderate
Lo Giudice 2024 [38]	SRMA of RCTs	6	Obese men and infertile men	1,297	340	PA	Aerobic training, resistance training, physical activity	150–240 min/week	12–24 weeks	-	3, 5, 49, 50, 53, 56, 57	Low risk	Moderate
Caldwell 2024 [39]	SRMA of RCTs	16	Obese women	3,588	-	EI/PA	Physical activity	-	4–52 weeks	DI, PI	3, 5, 7	Low risk	Moderate
Ruiz-González 2024 [40]	SRMA-NMA of RCTs	95	Obese women	9,910	-	EI	Resistance training, aerobic training, physical activity, moderate-intensity exercise	96–280 min/week	10–32 weeks	DI, PI	2, 20, 27, 28, 29, 30, 31, 33, 36, 56, 57	Low risk	Moderate
Song 2025 [41]	SRMA-NMA of RCTs	14	Fertile men or non-congenitally infertile men	-	1,079	EI	Outdoor aerobic, indoor aerobic, resistance training, Multi-component motion, competitive sports, bicycle aerobics, aerobic endurance exercise	120–900 min/week	4–52 weeks	-	48, 49, 55, 58, 59, 60, 61	Low risk	Moderate

AMH – anti-Müllerian hormone, BMI – body mass index, BP – blood pressure, DHEA-S – dehydroepiandrosterone sulfate, DI – dietary intervention, E2 – estradiol, EI – exercise intervention, EPA – elite physical activity, FAI – free androgen index, FG – Ferriman-Gallwey score, FI – fasting insulin, FPG – fasting plasma glucose, FSH – follicle-stimulating hormone, HDL-C – high-density lipoprotein cholesterol, HOMA-IR – homeostatic model assessment for insulin resistance, HR – heart rate, LDL-C – low-density lipoprotein cholesterol, LH – luteinizing hormone, PA – physical activity, PBA – psychological/behavioural intervention, PCOS – polycystic ovary syndrome, PI – pharmacological intervention, RCTs – randomised controlled trials, SHBG – sex hormone-binding globulin, SRMA – systematic review and meta-analysis, SRMA-MR – systematic review, meta-analysis, and meta-regression, SRMA-NMA – systematic review, meta-analysis, and network meta-analysis, T – testosterone, TC – total cholesterol, TG – triglycerides, VO2max – maximal oxygen uptake, WHR – waist-to-hip ratio

*Outcomes: 1. Weight; 2. Body mass index; 3. Natural pregnancy rate; 4. Clinical pregnancy; 5. Live birth; 6. Birth weight; 7. Miscarriage; 8. Pre-eclampsia; 9. Gestational diabetes; 10. Neonatal mortality; 11. Anthropometric measures; 12. Metabolic profiles; 13. Quality of life; 14. Mood; 15. Waist-to-hip ratio; 16. Fasting insulin; 17. Homeostatic model assessment for insulin resistance; 18. Low-density lipoprotein cholesterol; 19. High-density lipoprotein cholesterol; 20. Sex hormone-binding globulin; 21. Fasting plasma glucose; 22. Total cholesterol; 23. Triglycerides; 24. Blood pressure; 25. Heart rate; 26. Ferriman-Gallwey score; 27. Dehydroepiandrosterone sulfate; 28. Testosterone; 29. Follicle-stimulating hormone; 30. Luteinizing hormone; 31. Estradiol; 32. Menstrual cycle; 33. Ovulation rate; 34. Fertility; 35. Waist circumference; 36. Free androgen index; 37. Implantation rate; 38. Fertilisation rate; 39. Ongoing pregnancy; 40. Seminal oxidative stress markers; 41. Seminal inflammation markers; 42. Body composition; 43. Maximal oxygen uptake; 44. Anti-Müllerian hormone levels; 45. Lipid profile; 46. Insulin sensitivity; 47. Sperm concentration; 48. Sperm total motility; 49. Sperm forward motility; 50. Sperm quality; 51. Sperm count; 52. Glucose; 53. Insulin; 54. Semen volume; 55. Normal morphology; 56. Androstenedione; 57. Progesterone; 58. Sperm density; 59. Number of active spermatozoa; 60. Number of necrotic spermatozoa; 61. Infertility risk.

### Effects of exercise on female reproductive health and fertility capacity

#### Circulating sex hormone profiles

**Figure 2:**
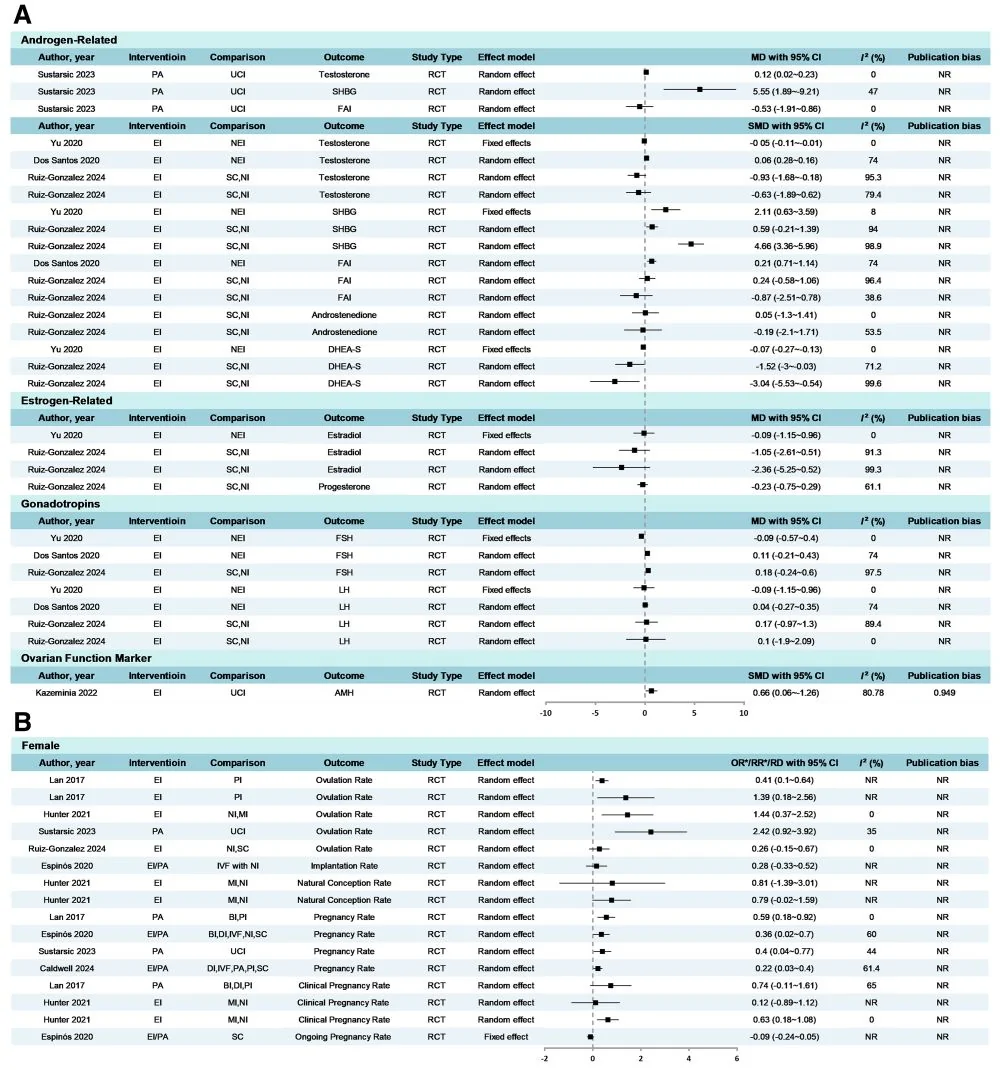
Summary of exercise interventions for female reproductive health and fertility capacity. Panel A. Hormonal parameters. Panel B. Fertility capacity and pregnancy rate. Effect sizes are shown with corresponding 95% confidence intervals, with *I*^2^ values indicating heterogeneity. *Log-transformed. AMH – anti-Müllerian hormone, BI – behavioural intervention, DHEA-S – dehydroepiandrosterone sulfate, DI – dietary intervention, EI – exercise intervention, FAI – free androgen index, FSH – follicle-stimulating hormone, IA – indoor aerobics, IVF – *in vitro* fertilisation, LH – luteinizing hormone, MD – mean difference, MI – minimal intervention, NEI – non-exercise intervention, NI – no intervention, NR – not reported, OA – outdoor aerobics, OR – odds ratio, PA – physical activity, PI – pharmacological intervention, RCT – randomised controlled trial, RD – risk difference, RR – risk ratio, SC – standard care, SHBG – sex hormone-binding globulin, SMD – standardised mean difference, UCI – undefined control intervention.

Four meta-analyses of RCTs including 6789 participants evaluated exercise and circulating sex hormones [30,31,37,40] (**Figure 2**, Panel A), with certainty ranging from high to very low (Table S4 in the **Online Supplementary Document**). Among these, one study reported a reduction in DHEA-S levels compared with control conditions comprising standard care or no intervention (SMD = −3.04; 95% CI = −5.53, −0.54) [40]. Another meta-analysis enrolling 273 participants reported that exercise significantly decreased AMH levels in women with PCOS compared with controls (SMD = 0.66; 95% CI = 0.06, 1.26) [35]. In addition, compared with the control group, female participants after exercise training demonstrated significantly higher levels of SHBG (MD = 5.55; 95% CI = 1.89, 9.21) and testosterone (MD = 0.12; 95% CI = 0.02, 0.23) [37]. By contrast, no significant improvement was observed in serum levels of FAI [37,40], androstenedione [40], oestrogens [30,40], or gonadotropins between exercise and control groups [30,31,40]. Narrative sensitivity analyses by population and intervention type showed patterns broadly consistent with the primary synthesis (Figures S1 and S2 in the **Online Supplementary Document**). In women with PCOS, positive changes in SHBG, AMH, and DHEA-S were reported in exercise group [30,31,35]. In women with obesity, there were obvious alterations in terms of DHEA-S and SHBG [40]. By intervention type, sole exercise interventions largely mirrored the main findings, whereas exercise combined with co-interventions showed larger but more variable patterns [37,40].

#### Ovulation and pregnancy outcomes

For ovulation and pregnancy-related outcomes, GRADE certainty ranged from high to very low, and selected favourable estimates were supported by moderate- or high-certainty evidence (Table S4 in the **Online Supplementary Document**). Four RCT meta-analyses including 609 participants assessed ovulation [29,33,37,40]. Exercise was associated with increased ovulation rates compared with control conditions (log-transformed OR (log-OR) = 2.42; 95% CI = 0.92, 3.92) [37], suggesting a promising effect on female fertility (**Figure 2**, Panel B). Six meta-analyses including 8252 participants evaluated pregnancy outcomes [29,32,33,37–39]. Pooled results showed increased pregnancy rates in the intervention group compared with the control group receiving behavioural or pharmacological interventions (log-OR = 0.59; 95% CI = 0.18, 0.92) [37], and clinical pregnancy rates were also higher than those receiving minimal or no intervention (log-transformed RR (log-RR) = 0.63; 95% CI = 0.18, 1.08) [29,33]. Additionally, two meta-analyses involving 120 participants assessed the effects of exercise on implantation and natural conception rates [32,33]. There was no significant improvement in implantation rates after intervention compared to the group receiving IVF without previous interventions (log-RR = 0.28; 95% CI = −0.33, 0.52) [32]. Similarly, natural conception rates showed no significant improvement compared with the control group receiving no or minimal intervention (log-RR = 0.81; 95% CI = −1.39, 3.01) [33]. Notably, these findings were observed among women with obesity [29,32,33,37–39].

### Effects of exercise on male reproductive health and fertility capacity

#### Sperm quality

**Figure 3:**
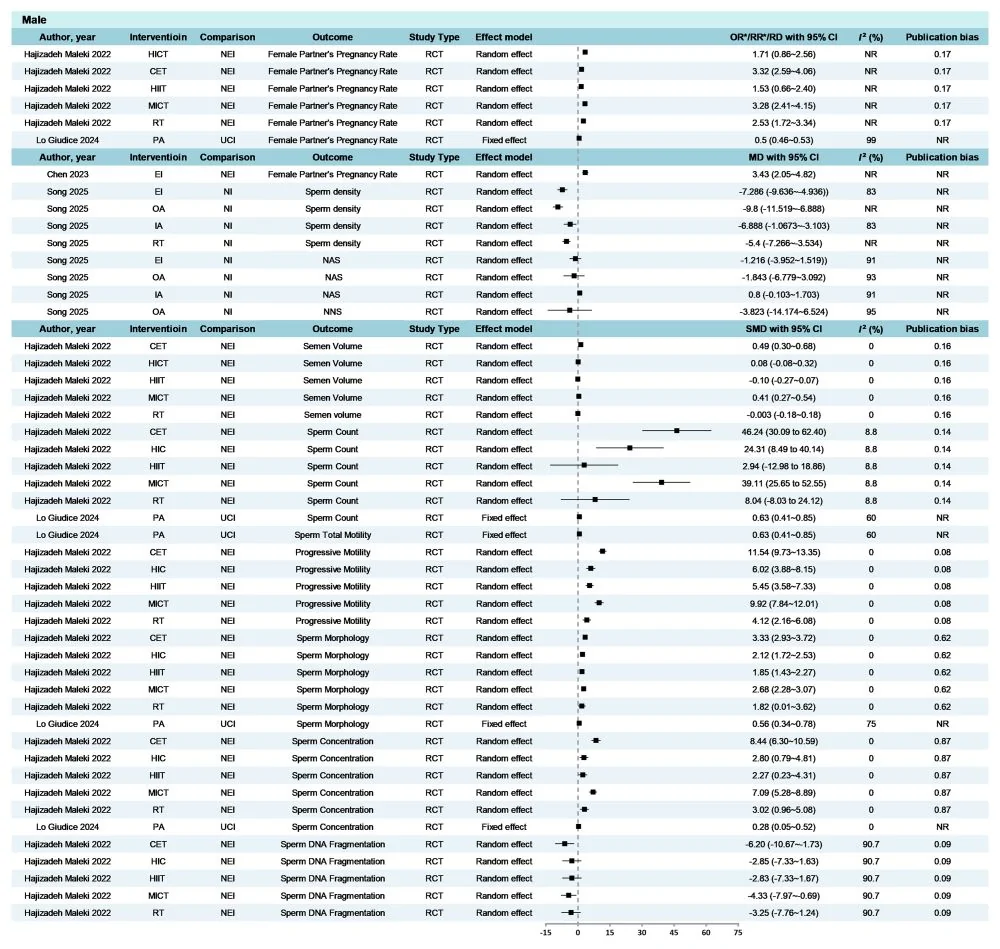
Summary of exercise training for male fertility capacity. The effects of exercise training on sperm quality, including concentration, volume, motility, count, morphology, DNA fragmentation, and pregnancy rates in female partners of males undergoing exercise interventions across various exercise modalities. Effect sizes are shown with corresponding 95% confidence intervals, with *I*^2^ values indicating heterogeneity. *Log-transformed. CET – combined aerobic and resistance training, EI – exercise intervention, HICT – high-intensity continuous training, HIIT – high-intensity interval training, IPA before IVF – inactive physical activity before *in vitro* fertilisation, MD – mean difference, MICT – moderate-intensity continuous training, NAS – number of active spermatozoa, NEI – non-exercise intervention, NI – no intervention, NNS – number of necrotic spermatozoa, NR – not reported, OR – odds ratio, PA – physical activity, RCT – randomised controlled trial, RD – risk difference, RR – risk ratio, RT – resistance training, SMD – standardised mean difference, UCI – undefined control intervention.

Three meta-analyses of RCTs including 5017 participants evaluated the effects of exercise on semen quality [34,38,41] (**Figure 3**), with GRADE certainty ranging from moderate to low (Table S4 in the **Online Supplementary Document**). All cardiorespiratory and resistance exercise modalities, including CET, MICT, HICT, HIIT, RT, exercise was associated with improved semen quality *vs.* non-exercise group [34,38]. The most pronounced benefits were observed with CET, including progressive motility (SMD = 11.54; 95% CI = 9.73, 13.35), sperm concentration (SMD = 8.44; 95% CI = 6.30, 10.59), sperm morphology (SMD = 3.33; 95% CI = 2.93, 3.72), semen volume (SMD = 0.49; 95% CI = 0.30, 0.68), and reduced sperm DNA fragmentation (SMD = −6.20; 95% CI = −10.67, −1.73) [34]. In addition, even in participants subjected to PA, there was enhanced sperm concentration (SMD = 0.28; 95% CI = 0.05, 0.52), semen volume (SMD = 0.15; 95% CI = 0.06, 0.36), sperm morphology (SMD = 0.56; 95% CI = 0.34, 0.78), semen count (SMD = 0.62; 95% CI = 0.19, 1.05), and total motility (SMD = 0.63; 95% CI = 0.41, 0.85) when compared with controls [38]. Narrative sensitivity analysis stratified by intervention type showed the pattern of findings for semen outcomes were primarily driven from exercise-only interventions (Figure S3 in the **Online Supplementary Document**) [34,38,41], indicating a potential specific direct effects of exercise on spermatogenic function and male reproductive health.

#### Partner pregnancy rates following exercise intervention in men

Two meta-analyses of RCTs involving 4642 male participants assessed pregnancy in female partners [34,36] (**Figure 3**), where the GRADE certainty was from low to very low (Table S4 in the **Online Supplementary Document**). Exercise intervention in men was associated with higher partner pregnancy rates than non-exercise controls (MD = 3.43; 95% CI = 2.05, 4.82) [36]. The highest estimated effect on pregnancy rates was observed for CET (log-RR = 3.32; 95% CI = 2.59, 4.06), followed by MICT (log-RR = 3.28; 95% CI = 2.41, 4.15) [34]. The narrative sensitivity analysis also suggested consistent beneficial patterns for exercise-only interventions, particularly CET and MICT, and exercise combined with other types of interventions (Figure S3 in the **Online Supplementary Document**) [34,36].

### Exercise modality-specific patterns in reproductive outcomes

To characterise whether exercise modalities have differential effects with female fertility outcomes, we visualised their connections using an alluvial diagram (Figure S4 in the **Online Supplementary Document**). Cardiorespiratory exercise was associated with higher ovulation, pregnancy, and clinical pregnancy rates, as well as hormonal outcomes, including higher SHBG, testosterone, and AMH levels and lower DHEA-S levels. Resistance training showed similar associations with ovulation and pregnancy rates and hormonal markers, with higher SHBG, testosterone, and AMH levels and lower DHEA-S levels. Structured exercise was linked to ovulation, pregnancy, and clinical pregnancy rates and was specifically connected to SHBG levels. In contrast, neuromotor training was linked only to AMH levels. Taken together, cardiorespiratory exercise, resistance training, and structured exercise may have broader reproductive benefits in females.

Similarly, we visualised exercise modality-outcome connections in males using an alluvial diagram (Figure S5 in the **Online Supplementary Document**). Cardiorespiratory exercise, including aerobic training, moderate-intensity continuous training, and high-intensity protocols, was the most broadly connected modality, being linked to all assessed outcomes, including sperm quality parameters, sperm concentration, progressive motility, sperm DNA fragmentation, semen volume, sperm motility, sperm count, sperm morphology, and female partners’ pregnancy rates. Resistance training and structured training also demonstrated substantial associations with improvements in semen parameters and partner pregnancy rates. Overall, our data suggested that cardiorespiratory exercise has the broadest effects for male reproductive health.

## DISCUSSION

Our study synthesis the latest evidence from systematic reviews and meta-analyses to reveal the effects of exercise interventions on reproductive health and fertility-related outcomes. In female, exercise was associated with beneficial changes in circulating sex hormone profiles, including SHBG, AMH, and DHEA-S, and was linked to higher ovulation rate, pregnancy rate and clinical pregnancy rate. In males, exercise was associated with enhanced sperm quality and linked to increased partner pregnancy rates. Collectively, these findings offer a clinical-based evidence of benefits of exercise of structured exercise in reproductive health.

Infertility has a multifactorial aetiology and can result from disruptions in either female or male reproductive physiology. Lifestyle factors, including smoking, recreational drug use, and obesity, are linked to reduced fertility [42]. In females, ovulatory disorders are a common cause of infertility, and anovulation is frequently associated with PCOS [43]. In males, potentially modifiable factors such as varicocele, obesity, and chronic disease have been associated with infertility [44]. Our findings support exercise as a protective lifestyle intervention, with durations ranging from 4 to 52 weeks, to ameliorate fertility-related outcomes. Across meta-analyses of randomised trials, exercise was associated with favourable directions of effect across multiple female and male endpoints, spanning both intermediate reproductive markers and selected reproductive outcomes. Collectively, these findings suggest that structured exercise may be associated with favourable reproductive outcomes within the broader multifactorial context of infertility.

Mechanistically, exercise may benefit reproductive health through several pathways. First, regular exercise training is known to reduce obesity, attenuate systemic inflammation, and improve metabolic homeostasis [45]. All of those improvements are critical for optimal reproductive health in both females and males [46,47]. Moreover, exercise may support hormonal homeostasis, a hallmark of reproductive health. For instance, elevated androgen levels disrupt aromatase activity and impair follicular development and ovulation [48,49], which contributes to anovulation and infertility in women with PCOS [50]. Our findings indicated that exercise training increases SHBG levels in females, which is positively associated with higher ovulation rates [51] and increased conception [52]. Higher testosterone levels were also observed, which may benefit fertility because low basal serum testosterone is linked to recurrent pregnancy loss [53]. Serum AMH level serve as a marker of the number of antral and pre-antral follicles [54]. Reductions in serum AMH levels were observed after exercise in patients with PCOS, suggesting that exercise may regulate ovarian function and support follicular development. In terms of male fertility, exercise can reverse reduced sperm motility and sperm DNA damage in obese male mice by attenuating ROS levels [55]. These findings indicate that exercise has the potential to improve testicular function and promote spermatogenesis. Additionally, exercise may enhance fertility by targeting the gut microbiome [56]. Exercise substantially promotes gut ecosystem diversity and fermentation capacity to produce metabolites with metabolic benefits [57,58]. For example, spermidine and L-citrulline are typical microbial-derived spermatogenesis factors [59] with positive effects on fertilisation efficiency. Ex vivo and *in vivo* studies showed that spermidine or L-citrulline treatment significantly improves sperm quality [60,61]. In women, microbiome dysbiosis caused by antibiotic exposure or infertility-related reproductive disorders, such as PCOS and endometriosis, is associated with impaired fertility capacity [62]. The gut microbiome plays a pivotal role in regulating oestrogen and androgen biosynthesis [63], thereby directly promoting women’s reproductive health.

Of note, we further investigated the specific effects of different exercise modalities on fertility outcomes as visualised in the alluvial diagram. In females, exercise interventions, including cardiorespiratory exercise, resistance training, structured exercise, and neuromotor training, were associated with improved ovulation and pregnancy outcomes, along with beneficial changes in hormonal parameters. Among these modalities, cardiorespiratory exercise emerged as particularly effective in enhancing reproductive outcomes. Similarly, in men, cardiorespiratory exercise demonstrated the most pronounced benefits for sperm quality and female partners' pregnancy outcomes, while resistance training and structured exercise also showed positive effects [34,36]. Studies have shown that male fertility strongly depends on environmental factors and lifestyle choices, in which both immediate and long-term changes in daily habits can effectively improve sperm quality and fertility outcomes [64]. Contradictory findings emerged regarding the effects of exercise intensity and duration on male reproductive outcomes. Moderate-intensity physical activity has been associated with favorable changes in semen parameters, particularly sperm motility and morphology [65], as well as improved pregnancy rates in female partners [34,36]. However, relatively limited improvement in male reproductive health was observed after high-intensity training of prolonged duration [34]. Overall, our umbrella review suggests that exercise with appropriate intensity, regardless of training method, may improve a broad spectrum of reproductive outcomes in both sexes.

Building on the relationship between exercise and reproductive outcomes, energy balance may represent an important intermediary factor in this interplay. Energy balance refers to the equilibrium between energy intake through diet and energy expenditure through physical activity and basal metabolic processes [66]. Disruptions in energy balance, particularly negative energy balance caused by excessive energy expenditure without adequate caloric intake, can lead to hormonal dysregulation and impaired reproductive function [67]. Negative energy balance has been linked to functional hypothalamic amenorrhea in women [68] and to reduced testosterone levels [69] and impaired spermatogenesis in men [70]. While energy balance is fundamental, exercise intensity also plays a significant role because of its direct effect on energy expenditure [71]. Evidence shows that intense physical work can suppress ovarian function even in the absence of nutritional stress [72], suggesting that exercise intensity may exert independent effects on reproductive health. Moreover, studies in metabolic health reveal that high-intensity and moderate-intensity exercise, even when matched for energy expenditure, can produce distinct physiological outcomes [73,74]. However, it remains unclear how different exercise intensities, when matched for energy expenditure and energy balance, affect reproductive health. Future research should clarify the relationships among exercise modalities, exercise intensity, energy balance, and reproductive outcomes.

To the best of our knowledge, our study provides the most up-to-date umbrella review evaluating the potential effects of exercise-based interventions on fertility-related outcomes in both sexes. Exercise exerts beneficial effects on hormone profiles and ovulatory function in females, as well as enhanced sperm quality in males. However, several limitations should be acknowledged. First, the included meta-analyses exhibited considerable heterogeneity in exercise protocols in terms of duration, intensity, frequency, and modality, as well as across participant populations, limiting our ability to determine the optimal exercise prescription to support fertility. The certainty of evidence ranged from high to very low across outcomes, with most evidence rated as moderate, low, or very low certainty, which reduces confidence in the observed associations. Second, clinical heterogeneity across the included meta-analyses may limit interpretation of the reported pooled estimates. Differences in infertility phenotypes, metabolic status, comorbidities, and exercise protocols may have increased between-review variability and reduced the applicability of findings to specific clinical subgroups. The forest plots summarise review-level outcome comparisons rather than newly pooled or statistically independent meta-analyses and should therefore be interpreted as a descriptive overview of the direction and magnitude of evidence across reviews. Third, 7 of the 13 included meta-analyses incorporated co-interventions alongside exercise, such as dietary interventions, pharmacological interventions, and psychological or behavioural advice. These co-interventions may have contributed to between-review variability and were therefore considered in sensitivity analyses where possible. These additional analyses helped assess the consistency of the findings across intervention contexts, while future studies may further refine estimates of the specific contribution of exercise to fertility outcomes. Moreover, definitions of exercise intensity and exercise modalities were often unclear or overlapping, both within the literature and in clinical practice. The lack of detailed information hindered dose-response analyses and comparisons of the efficacy of exercise for fertility outcomes. Additionally, incomplete reporting of participants’ baseline reproductive status and demographics increases the risk of selection bias and reduces the external validity of our conclusions. Lastly, there is a lack of direct comparisons across different exercise modalities within the same study. Collectively, these limitations highlight the need for future research to evaluate exercise as a standalone intervention, incorporate prespecified subgroup analyses, and ensure sufficiently detailed intervention reporting to better determine the independent impact of exercise on fertility outcomes. Future studies should also include direct comparisons of exercise modalities to clarify modality-specific effects on reproductive outcomes and support the development of more targeted exercise regimens.

## CONCLUSIONS

Taken together, our results suggest that exercise is potentially associated with improvements in reproductive health outcomes. It was linked to positive changes in hormone profiles, enhanced ovulation and pregnancy rates in females, and improved sperm quality in male, implying exercise may represent an accessible and non-pharmaceutical adjunctive strategy in fertility management. However, the overall confidence in current evidence remains constrained, with most rated as moderate, low, or very low certainty. Further high-quality trials are needed to clarify optimal exercise prescriptions and strengthen the certainty of evidence.

## Additional material

Online Supplementary Document

## Data Availability

**Data availability:** All relevant data addressed in the manuscript can be obtained from the corresponding author upon reasonable request.
