# Supplementary material for: Exercise intervention improves fertility outcomes in females and males: an umbrella review of meta-analyses
Source: J Glob Health. 2026 Aug 28;16:04282. doi: 10.7189/jogh-16-04282 (PMC13522903; doi:10.7189/jogh-16-04282)
Supplement: Supplementary file 1 [file jogh-16-04282-s001.pdf]

Supplement to: Lam LK, Alemu BK, Davenport MH, Wang CC, Chan YL, Wang Y. Exercise intervention improves fertility outcomes in females and males: an umbrella review of meta-analyses. J Glob Health. 2026;16:04282.

Table S1. Searching terms and outputs for six databases

Database	Terms	Result
Pubmed	(("Exercise" OR "exercises" OR "exercise therapy" OR ("exercise" AND "therapy") OR "exercising" OR "exercise s" OR "exercised" OR "exerciser" OR "exercisers" OR "Physical Exercise" OR "Aerobic Exercise" OR "Exercise Training" OR "Physical Activity") AND ("womans" OR "women" OR "woman" OR "women s" OR "womens" OR "girl" OR "men" OR "boys") AND ("fertiles" OR "fertility" OR "fertile" OR "fertilities" OR "fecund" OR "fecundities" OR "fecundity" OR "infertiles" OR "infertilities" OR "infertility" OR "infertile" OR "infertility s" OR "sterile" OR "sterility" OR "sterilant" OR "sterilants" OR "sterilely" OR "steriles" OR "sterilisation" OR "sterilization, reproductive" OR ("sterilization" AND "reproductive") OR "reproductive sterilization" OR "sterilization" OR "sterilisations" OR "sterilised" OR "steriliser" OR "sterilisers" OR "sterilising" OR "sterilities" OR "sterilise" OR "sterilize" OR "sterilizations" OR "sterilized" OR "sterilizer" OR "sterilizers" OR "sterilizes" OR "sterilizing")) AND ("Systematic review and meta-analysis" OR "review" OR "meta-analysis"))	422
Web of Science	TS=(("exercise" OR "physical activity" OR "sport" OR "training" OR "workout" OR "aerobic" OR "resistance" OR "strength training")) AND TS=(fertility OR infertility) AND TS=("systematic review" OR "meta-analysis" OR "systematic review and meta-analysis")	499
Cochrane Library	(((Exercise OR "breathing exercise" OR "physical activity") AND (women OR men) AND ("randomised control trials" OR "randomized controlled trials" OR "randomized controlled trial" OR randomized OR controlled) AND ("meta analysis" OR "meta-analysis") AND (fertility OR infertility)) OR ("cohort study" OR "case-control study" OR "cross sectional study" OR observational))	506
Scopus	TITLE-ABS-KEY("exercise" OR "physical activity" OR sport* OR training OR workout OR aerobic* OR resistance OR endurance OR "high-intensity" OR "strength training") AND TITLE-ABS-KEY(fertility OR infertility) AND TITLE-ABS-KEY("systematic review" OR "meta-analysis" OR "systematic review and meta-analysis") AND TITLE-ABS-KEY(women OR female* OR men OR male*) AND NOT TITLE-ABS-KEY("umbrella review" OR "comprehensive review" OR "narrative review" OR "literature review")	543
EBSCOhost	(Exercise OR "physical fitness" OR "physical activity") AND (women OR men OR female OR male) AND ("meta-analysis" OR "meta analysis" OR metanalysis) AND (fertility OR infertility OR reproductive OR fertile)	81
Google scholar	("Exercise" OR "breathing exercise" OR "physical activity") AND ("women" OR "men") AND ("meta-analysis" OR "meta analysis" OR "metanalysis" OR "meta") AND ("fertility" OR "infertility" OR "reproductive" OR "fertile") AND NOT ("narrative" OR "case") in Chinese website	275

Table S2. Graphical crosstabulation of the results of corrected covered area

	Lam 2017	Yu 2020	Dos Santos 2020	Espinós 2020	Hunter 2021	Hajizadeh 2022	Kazeminia 2022	Chen 2023	Sustarsic 2023	Giudice 2024	Caldwell 2024	Ruiz-González 2024	Song 2025
De Jong-Potjer et al., 2006	■												
Lumley and Donohue, 2006	■												
Hillemeier et al., 2008	■												
Downs et al., 2009	■												
Weisman et al., 2011	■												
Mutsaerts et al., 2010	■												
Mutsaerts et al., 2016	■			■	■				■		■	■	
Palomba et al., 2010	■			■	■				■				
Moran et al., 2011	■				■		■		■		■	■	
Sim et al., 2014	■			■	■				■		■	■	
Legro et al., 2015	■				■				■		■		
Dokras et al., 2016	■								■				
Almenning et al., 2015		■	■									■	
Elisabet et al., 2012		■											
Elizabeth et al., 2011		■											
Elisabet et al., 2009		■											
Giallauria et al., 2008		■	■										
Orio et al., 2016		■											
Vigorito et al., 2007		■											
Costa et al., 2018			■										
Gaeini et al., 2014			■										
Jedel et al., 2011			■										
Lopes et al., 2018			■										
Ribeiro et al., 2019			■										
Turan et al., 2015			■										
Vizza et al., 2016			■										
Becker et al., 2015				■	■				■		■	■	
Einarsson et al., 2017				■	■				■		■	■	
Espinós et al., 2017				■	■				■		■	■	
Moran et al., 2016				■									
Rothberg et al., 2016				■	■				■			■	
Duval et al., 2015					■								
Guzick et al., 1994					■							■	
Hoeger et al., 2004					■							■	
Kiel et al., 2018					■				■				
Nagelberg et al., 2016					■				■				
Nasrekani et al., 2016					■								
Hajizadeh Maleki et al., 2017						■		■	■	■			
Hajizadeh Maleki et al., 2020						■		■					
Hajizadeh Maleki et al., 2018						■							
Hajizadeh Maleki et al., 2019						■							
Saremi and Yaghoubi, 2016							■						
Saremi et al., 2013							■						
Saremi and Rajabi, 2016							■						
Saremi et al., 2014							■						
Al-Eisa et al., 2017							■						
Vosnakis et al., 2013							■						
Hosseini et al., 2019							■						
Nybacka et al., 2013							■					■	
Wu et al., 2021							■						
Leonhardt et al., 2015							■						
Nidhi et al., 2013							■						
Ketabchi et al., 2018								■					
Sun et al., 2016								■					
Jin et al., 2017								■					
Yu et al., 2019								■					
Nadjarzadeh et al., 2012								■					
Balercia et al., 2009								■					
Safarinejad et al., 2012								■					
Lenzi et al., 2004								■					
Balercia et al., 2005								■					
Rolf et al., 1999								■					
Kessopoulou et al., 1995								■					
Greco et al., 2005								■					
Haghighian et al., 2015								■					
Omu et al., 1997								■					
Ebisch et al., 2005								■					

Wong et al., 2002													
Raigani et al., 2013													
Martínez-Soto et al., 2016													
Safarinejad et al., 2009													
Safarinejad et al., 2008													
Scott et al., 1998													
Hawkes et al., 2009													
Nouri et al., 2019													
Behzad et al., 2017													
Galletly et al., 1996													
Thomson et al., 2008													
van Dammen et al., 2018													
Andersen et al., 2022													
Montano et al., 2022													
Rosety-Rodriguez et al., 2014													
Jiskoot et al., 2021													
LeBlanc et al., 2021													
Muirhead et al., 2021													
Price et al., 2020, 2021													
Rönö et al., 2018													
Rothberg et al., 2018													
Legro et al., 2016													
Legro et al., 2022													
Salamun et al., 2018													
Wang et al., 2021													
Aroda et al., 2020													
Begum et al., 2014													
Bonakdaran et al., 2012													
Bougoulia et al., 2006													
Brown et al., 2009													
Bruner et al., 2006													
Chou et al., 2003													
Crave et al., 1995													
Curi et al., 2012													
Elkind-Hirsch et al., 2008													
Fleming et al., 2002													
Florakis et al., 2007													
Frossing et al., 2018													
Gambineri et al., 2006													
Ghandi et al., 2011													
Jensterle et al., 2015													
Legro et al., 2007													
Lim et al., 2011													
Arce et al., 1993													
M. J De Souza et al., 1994													
Xiaolin Zhang et al., 2019													

Table S3. Graphical summary of AMSTAR 2 methodological quality assessment

Study	(1) PICO included	(2) Protocol established before review	(3) Study designs explained	(4) Comprehensive literature search	(5) Study selection in duplicate	(6) Data extraction in duplicate	(7) Excluded studies listed and justified	(8) Included studies described in adequate detail	(9) RoB assessment of included studies adequate	(10) Funding sources of included studies reported	(11) Appropriate meta-analysis methods used	(12) Impact of RoB on meta-analysis assessed	(13) RoB considered in interpretation/discussion	(14) Heterogeneity explained	(15) Publication bias investigated and discussed*	(16) Conflict of interest reported	Critical methodological concerns	Other methodological limitations	Overall confidence rating
Lan et al. 2017																	Partial: Items 2, 7, 15	Items 3, 10	Moderate
Yu et al. 2020																	Partial: Items 2, 4, 7, 13, 15	Items 3, 6, 10, 12, 14, 16	Moderate
Dos Santos et al. 2020																	Partial: Items 7, 15	Items 10, 12	Moderate
Espinós et al. 2020																	None identified	Item 12	High
Hunter et al. 2021																	Partial: Items 2, 4, 7, 13, 15	Items 5, 10, 12	Moderate
Hajizadeh Maleki et al. 2022																	Partial: Items 2, 7	Items 10, 12, 16	Moderate
Kazeminia et al. 2022																	Partial: Items 2, 7	Items 3, 10, 12	Moderate
Chen et al. 2023																	Partial: Items 2, 7, 13, 15	Items 10, 12, 14	Moderate
Sustarsic et al. 2023																	Partial: Items 2, 4, 7, 13, 15	Items 5, 6, 10, 12	Moderate
Lo Giudice et al. 2024																	Partial: Items 4, 7, 11, 13, 15	Items 6, 10, 12, 14	Moderate
Caldwell et al. 2024																	Partial: Items 4, 7, 15	Items 10, 12	Moderate
Ruiz-González et al. 2024																	Partial: Items 2, 4, 7, 15	Items 5, 6, 10, 12, 16	Moderate
Song et al. 2025																	Partial: Items 4, 7, 15	Items 5, 10, 12, 16	Moderate

Table S4. Outcome-level certainty of evidence assessed using the GRADE approach

Author, year	Outcome	Certainty assessment							No. of participants		Effect		Certainty
		No. of primary studies contributing to the outcome	Primary study designs in included meta-analyses	Risk of bias	Inconsistency	Indirectness	Imprecision	Publication bias	Experimental group	Control group	Relative (95% CI)	Absolute (95% CI)	
Lan et al. 2017	Pregnancy rate	2	RCTs	Serious	Not serious	Not serious	Not serious	Not assessable[a]	307	306	-	OR 1.87 (1.24~2.81)	⊕⊕⊕○ Moderate
	Clinical pregnancy rate	5	RCTs	Serious	Not serious	Not serious	Serious	Not assessable[a]	400	403	-	OR 2.10, (0.90~5.01)	⊕⊕○○ Low
	Ovulation rate	1	RCTs	Not serious	Not serious	Not serious	Not serious	Not assessable[a]	50	49	-	RR 1.5 (1.1~1.9)	⊕⊕⊕⊕ High
	Ovulation rate	1	RCTs	Not serious	Not serious	Not serious	Serious	Not assessable[a]	32	32	-	RR 4.0 (1.2~12.8)	⊕⊕⊕○ Moderate
Yu et al. 2032	DHEA-S	6	RCTs	Serious	Not serious	Not serious	Serious	Not assessable[a]	189	187	-	MD −0.07 (−0.27~0.13)	⊕⊕○○ Low
	SHBG	6	RCTs	Serious	Not serious	Not serious	Not serious	Not assessable[a]	189	187	-	MD 2.11 (0.63~3.59)	⊕⊕○○ Low
	Testosterone	4	RCTs	Serious	Not serious	Not serious	Serious	Not assessable[a]	151	163	-	MD −0.05 (0.11~0.01)	⊕⊕○○ Low
	FSH	4	RCTs	Serious	Not serious	Not serious	Serious	Not assessable[a]	151	163	-	MD −0.09 (−0.57~0.40)	⊕⊕○○ Low
	LH	4	RCTs	Serious	Not serious	Not serious	Serious	Not assessable[a]	151	163	-	MD −0.09 (−1.15~0.96)	⊕⊕○○ Low
	Estradiol	4	RCTs	Serious	Not serious	Not serious	Serious	Not assessable[a]	151	163	-	MD −0.60 (−7.45~6.25)	⊕⊕○○ Low
Dos Santos et al. 2020	FAI	3	RCTs	Serious	Serious	Not serious	Serious	Not assessable[a]	115	95	-	SMD 0.21 (−0.71~1.14)	⊕○○○ Very low
	FSH	2	RCTs	Serious	Serious	Not serious	Serious	Not assessable[a]	92	77	-	SMD 0.04 (−0.27~0.35)	⊕○○○ Very low
	LH	2	RCTs	Serious	Serious	Not serious	Serious	Not assessable[a]	92	77	-	SMD 0.11 (−0.21~0.43)	⊕○○○ Very low
	Testosterone	5	RCTs	Serious	Serious	Not serious	Serious	Not assessable[a]	202	140	-	SMD 0.06 (0.28~0.16)	⊕○○○ Very low
Espinós et al. 2020	Pregnancy rate	8	RCTs	Serious	Serious	Not serious	Serious	Not assessable[a]	550	548	-	RR 1.43 (1.02~2.01)	⊕○○○ Very low
	Ongoing pregnancy	1	RCT	Serious	Not serious	Not serious	Serious	Not assessable[a]	Total: 317	Total: 317	-	RR 0.91 (0.79~1.05)	⊕⊕○○ Low
	Implantation rate	1	RCT	Serious	Not serious	Not serious	Serious	Not assessable[a]	Total: 65	Total: 65	-	RR 1.32 (0.72~1.69)	⊕⊕○○ Low
Hunter et al. 2021	Clinical pregnancy rate	4	RCTs	Serious	Not serious	Not serious	Not serious	Not assessable[a]	130	128	-	RR 1.87 (1.20~2.93)	⊕⊕⊕○ Moderate
	Clinical pregnancy rate	1	RCT	Serious	Not serious	Not serious	Serious	Not assessable[a]	8	8	-	RR 1.13 (0.41~3.08)	⊕⊕○○ Low
	Ovulation rate	2	RCTs	Serious	Not serious	Not serious	Serious	Not assessable[a]	37	36	-	RR 4.24 (1.45~12.39)	⊕⊕○○ Low
	Natural conception rate	5	RCTs	Serious	Serious	Not serious	Not serious	Not assessable[a]	492	499	-	RR 2.25 (1.42~3.59)	⊕⊕○○ Low
	Natural conception rate	2	RCTs	Serious	Not serious	Not serious	Serious	Not assessable[a]	304	315	-	RR 2.20 (0.98~4.93)	⊕⊕○○ Low
Hajizadeh Maleki et al. 2022	Semen Volume	2	RCTs	Serious	Not serious	Not serious	Not serious	Not assessable[a]	Total: 651	Total: 651	-	SMD 0.49 (0.30~0.68)	⊕⊕⊕○ Moderate
	Semen Volume	2	RCTs	Serious	Not serious	Not serious	Serious	Not assessable[a]	Total: 520	Total: 520	-	SMD 0.08 (-0.08~0.32)	⊕⊕○○ Low
	Semen Volume	2	RCTs	Serious	Not serious	Not serious	Serious	Not assessable[a]	Total: 553	Total: 553	-	SMD -0.10 (-0.27~0.07)	⊕⊕○○ Low
	Semen Volume	3	RCTs	Serious	Not serious	Not serious	Not serious	Not assessable[a]	Total: 650	Total: 650	-	SMD 0.41 (0.27~0.54)	⊕⊕⊕○ Moderate
	Semen Volume	3	RCTs	Serious	Not serious	Not serious	Serious	Not assessable[a]	Total: 534	Total: 534	-	SMD -0.003 (-0.18~0.18)	⊕⊕○○ Low
	Progressive Motility	2	RCTs	Serious	Not serious	Not serious	Not serious	Not assessable[a]	Total: 651	Total: 651	-	SMD 11.54 (9.73~13.35)	⊕⊕⊕○ Moderate
	Progressive Motility	2	RCTs	Serious	Not serious	Not serious	Serious	Not assessable[a]	Total: 520	Total: 520	-	SMD 6.02 (3.88~8.15)	⊕⊕○○ Low
	Progressive Motility	2	RCTs	Serious	Not serious	Not serious	Serious	Not assessable[a]	Total: 553	Total: 553	-	SMD 5.45 (3.58~7.33)	⊕⊕○○ Low
	Progressive Motility	3	RCTs	Serious	Not serious	Not serious	Not serious	Not assessable[a]	Total: 650	Total: 650	-	SMD 9.92 (7.84~12.01)	⊕⊕⊕○ Moderate
	Progressive Motility	2	RCTs	Serious	Not serious	Not serious	Serious	Not assessable[a]	Total: 534	Total: 534	-	SMD 4.12 (2.16~6.08)	⊕⊕○○ Low
	Sperm Morphology	2	RCTs	Serious	Not serious	Not serious	Not serious	Not assessable[a]	Total: 651	Total: 651	-	SMD 3.33 (2.93~3.72)	⊕⊕⊕○ Moderate
	Sperm Morphology	2	RCTs	Serious	Not serious	Not serious	Not serious	Not assessable[a]	Total: 520	Total: 520	-	SMD 2.12 (1.72~2.53)	⊕⊕⊕○ Moderate
	Sperm Morphology	2	RCTs	Serious	Not serious	Not serious	Not serious	Not assessable[a]	Total: 553	Total: 553	-	SMD 1.85 (1.43~2.27)	⊕⊕⊕○ Moderate
	Sperm Morphology	3	RCTs	Serious	Not serious	Not serious	Not serious	Not assessable[a]	Total: 650	Total: 650	-	SMD 2.68 (2.28~3.07)	⊕⊕⊕○ Moderate
	Sperm Morphology	2	RCTs	Serious	Not serious	Not serious	Serious	Not assessable[a]	Total: 534	Total: 534	-	SMD 1.82 (0.01~3.62)	⊕⊕○○ Low

	Sperm Concentration	2	RCTs	Serious	Not serious	Not serious	Not serious	Not assessable[a]	Total: 651	Total: 651	-	SMD 8.44 (6.30~10.59)	⊕⊕⊕○ Moderate
	Sperm Concentration	2	RCTs	Serious	Not serious	Not serious	Serious	Not assessable[a]	Total: 520	Total: 520	-	SMD 2.80 (0.79~4.81)	⊕⊕○○ Low
	Sperm Concentration	2	RCTs	Serious	Not serious	Not serious	Serious	Not assessable[a]	Total: 553	Total: 553	-	SMD 2.27 (0.23~4.31)	⊕⊕○○ Low
	Sperm Concentration	3	RCTs	Serious	Not serious	Not serious	Not serious	Not assessable[a]	Total: 650	Total: 650	-	SMD 7.09 (5.28~8.89)	⊕⊕⊕○ Moderate
	Sperm Concentration	2	RCTs	Serious	Not serious	Not serious	Serious	Not assessable[a]	Total: 534	Total: 534	-	SMD 3.02 (0.96~5.08)	⊕⊕○○ Low
	Sperm DNA Fragmentation	2	RCTs	Serious	Serious	Not serious	Serious	Not assessable[a]	Total: 651	Total: 651	-	SMD -6.20 (-10.67~-1.73)	⊕○○○ Very low
	Sperm DNA Fragmentation	2	RCTs	Serious	Serious	Not serious	Serious	Not assessable[a]	Total: 520	Total: 520	-	SMD -2.85 (-7.33~1.63)	⊕○○○ Very low
	Sperm DNA Fragmentation	2	RCTs	Serious	Serious	Not serious	Serious	Not assessable[a]	Total: 553	Total: 553	-	SMD -2.83 (-7.33~1.67)	⊕○○○ Very low
	Sperm DNA Fragmentation	3	RCTs	Serious	Serious	Not serious	Serious	Not assessable[a]	Total: 650	Total: 650	-	SMD -4.33 (-7.97~-0.69)	⊕○○○ Very low
	Sperm DNA Fragmentation	2	RCTs	Serious	Serious	Not serious	Serious	Not assessable[a]	Total: 534	Total: 534	-	SMD -3.25 (-7.76~1.24)	⊕○○○ Very low
	Sperm Count	2	RCTs	Serious	Not serious	Not serious	Not serious	Not assessable[a]	Total: 651	Total: 651	-	SMD 46.24 (30.09 ~ 62.40)	⊕⊕⊕○ Moderate
	Sperm Count	2	RCTs	Serious	Not serious	Not serious	Serious	Not assessable[a]	Total: 520	Total: 520	-	SMD 24.31 (8.49 ~ 40.14)	⊕⊕○○ Low
	Sperm Count	2	RCTs	Serious	Not serious	Not serious	Serious	Not assessable[a]	Total: 553	Total: 553	-	SMD 2.94 (-12.98 ~ 18.86)	⊕⊕○○ Low
	Sperm Count	3	RCTs	Serious	Not serious	Not serious	Not serious	Not assessable[a]	Total: 650	Total: 650	-	SMD 39.11 (25.65 ~ 52.55)	⊕⊕⊕○ Moderate
	Sperm Count	2	RCTs	Serious	Not serious	Not serious	Serious	Not assessable[a]	Total: 534	Total: 534	-	SMD 8.04 (-8.03 ~ 24.12)	⊕⊕○○ Low
	Female Partner's Pregnancy Rate	2	RCTs	Serious	Not serious	Not serious	Serious	Not assessable[a]	Total: 651	Total: 651	-	RR 27.81 (13.34~57.97)	⊕⊕○○ Low
	Female Partner's Pregnancy Rate	2	RCTs	Serious	Not serious	Not serious	Serious	Not assessable[a]	Total: 520	Total: 520	-	RR 5.55 (2.37~13)	⊕⊕○○ Low
	Female Partner's Pregnancy Rate	2	RCTs	Serious	Not serious	Not serious	Serious	Not assessable[a]	Total: 553	Total: 553	-	RR 4.63 (1.94~11.02)	⊕⊕○○ Low
	Female Partner's Pregnancy Rate	3	RCTs	Serious	Not serious	Not serious	Serious	Not assessable[a]	Total: 650	Total: 650	-	RR 26.67 (11.18~63.63)	⊕⊕○○ Low
	Female Partner's Pregnancy Rate	2	RCTs	Serious	Not serious	Not serious	Serious	Not assessable[a]	Total: 534	Total: 534	-	RR 12.54 (5.58~28.19)	⊕⊕○○ Low
Kazeminia et al. 2022	Anti-Mullerian hormone level	6	RCTs	Serious	Serious	Not serious	Serious	Not assessable[a]	134	139	-	SMD 0.66 (0.06~1.26)	⊕○○○ Very low
Chen et al. 2023	Female Partner's Pregnancy Rate	4	RCTs	Serious	Serious	Not serious	Not serious	Not assessable[a]	Total: 1734	Total: 1734	-	MD 3.43 (2.05~4.82)	⊕⊕○○ Low
Sustarsic et al. 2023	SHBG	5	RCTs	Not serious	Not serious	Not serious	Not serious	Not assessable[a]	Total: 551	Total: 551	-	MD 5.55 (1.89~9.21)	⊕⊕⊕⊕ High
	FAI	3	RCTs	Not serious	Not serious	Not serious	Serious	Not assessable[a]	Total: 172	Total: 172	-	MD −0.53 (−1.91~0.86)	⊕⊕⊕○Moderate
	Testosterone	5	RCTs	Not serious	Not serious	Not serious	Not serious	Not assessable[a]	Total: 551	Total: 551	-	MD 0.12 (0.02~0.23)	⊕⊕⊕⊕ High
	Ovulation Rate	4	RCTs	Not serious	Not serious	Not serious	Serious	Not assessable[a]	Total: 123	Total: 123	-	OR 11.23 (2.51~50.23)	⊕⊕⊕○Moderate
	Pregnancy Rate	11	RCTs	Not serious	Not serious	Not serious	Not serious	Serious	Total: 1526	Total: 1526	-	OR 1.49 (1.04~2.15)	⊕⊕⊕○Moderate
Lo Giudice et al. 2024	Sperm Concentration	2	RCTs	Not serious	Not serious	Not serious	Not serious	Not assessable[a]	146	134	-	SMD 0.28 (0.05~0.52)	⊕⊕⊕⊕ High
	Semen volume	3	RCTs	Not serious	Not serious	Not serious	Serious	Not assessable[a]	176	164	-	SMD 0.15 (−0.06~0.36)	⊕⊕⊕○ Moderate
	Sperm Total Motility	3	RCTs	Not serious	Serious	Not serious	Not serious	Not assessable[a]	176	164	-	SMD 0.63 (0.41~0.85)	⊕⊕⊕○ Moderate
	Sperm Count	2	RCTs	Not serious	Not serious	Not serious	Serious	Not assessable[a]	39	60	-	SMD 0.62 (0.19~1.05)	⊕⊕⊕○ Moderate
	Sperm Morphology	2	RCTs	Not serious	Serious	Not serious	Not serious	Not assessable[a]	167	156	-	SMD 0.56 (0.34~0.78)	⊕⊕⊕○ Moderate
	Female Partner's Pregnancy Rate	3	RCTs	Not serious	Serious	Not serious	Not serious	Not assessable[a]	643	654	-	RD 0.50 (0.46~0.53)	⊕⊕⊕○ Moderate
Caldwell et al. 2024	Pregnancy Rate	12	RCTs	Serious	Serious	Not serious	Serious	Serious	Total: 1052	Total: 970	-	RR 1.24 (1.03~1.49)	⊕○○○ Very low
Ruiz-González et al. 2024	Ovulation rate	4	RCTs	Serious	Serious	Not serious	Serious	Not assessable[a]	Not reported by outcome	Not reported by outcome	-	RR 1.30 (0.86~1.96)	⊕○○○ Very low
	Total testosterone	13	RCTs	Serious	Serious	Not serious	Serious	Serious	Not reported by outcome	Not reported by outcome	-	SMD -0.93 (-1.68~-0.18)	⊕○○○ Very low
	Total testosterone	6	RCTs	Serious	Serious	Not serious	Serious	Not assessable[a]	Not reported by outcome	Not reported by outcome	-	SMD -0.63 (-1.89~0.62)	⊕○○○ Very low
	SHBG	12	RCTs	Serious	Serious	Not serious	Serious	Serious	Not reported by outcome	Not reported by outcome	-	SMD 0.59 (-0.21~1.39)	⊕○○○ Very low
	SHBG	7	RCTs	Serious	Serious	Not serious	Serious	Not assessable[a]	Not reported by outcome	Not reported by outcome	-	SMD 4.66 (3.36~5.96)	⊕○○○ Very low

	FAI	13	RCTs	Serious	Serious	Not serious	Serious	Serious	Not reported by outcome	Not reported by outcome	-	SMD 0.24 (-0.58~1.06)	⊕○○○ Very low
	FAI	3	RCTs	Serious	Not serious	Not serious	Serious	Not assessable[a]	Not reported by outcome	Not reported by outcome	-	SMD -0.87 (-2.51~0.78)	⊕⊕○○ Low
	Androstenedione	4	RCTs	Serious	Not serious	Not serious	Serious	Not assessable[a]	Not reported by outcome	Not reported by outcome	-	SMD 0.05 (-1.3~1.41)	⊕⊕○○ Low
	Androstenedione	2	RCTs	Serious	Serious	Not serious	Serious	Not assessable[a]	Not reported by outcome	Not reported by outcome	-	SMD -0.19 (-2.1~1.71)	⊕○○○ Very low
	FSH	6	RCTs	Serious	Serious	Not serious	Serious	Not assessable[a]	Not reported by outcome	Not reported by outcome	-	SMD 0.18 (-0.24~0.6)	⊕○○○ Very low
	LH	6	RCTs	Serious	Serious	Not serious	Serious	Not assessable[a]	Not reported by outcome	Not reported by outcome	-	SMD 0.17 (-0.97~1.3)	⊕○○○ Very low
	LH	2	RCTs	Serious	Not serious	Not serious	Serious	Not assessable[a]	Not reported by outcome	Not reported by outcome	-	SMD 0.1 (-1.9~2.09)	⊕⊕○○ Low
	Estradiol	7	RCTs	Serious	Serious	Not serious	Serious	Not assessable[a]	Not reported by outcome	Not reported by outcome	-	SMD -1.05 (-2.61~0.51)	⊕○○○ Very low
	Estradiol	2	RCTs	Serious	Serious	Not serious	Serious	Not assessable[a]	Not reported by outcome	Not reported by outcome	-	SMD -2.36 (-5.25~0.52)	⊕○○○ Very low
	Progesterone	2	RCTs	Serious	Serious	Not serious	Serious	Not assessable[a]	Not reported by outcome	Not reported by outcome	-	SMD -0.23 (-0.75~0.29)	⊕○○○ Very low
Song et al. 2025	Number of active spermatozoa	4	RCTs	Not serious	Serious	Not serious	Serious	Not assessable[a]	32	32	-	MD -12.16 (-39.52~15.19)	⊕⊕○○ Low
	Number of active spermatozoa	2	RCTs	Not serious	Serious	Not serious	Serious	Not assessable[a]	12	12	-	MD 18.43 (-67.79~30.92)	⊕⊕○○ Low
	Number of active spermatozoa	2	RCTs	Not serious	Serious	Not serious	Serious	Not assessable[a]	20	20	-	MD 8.00 (-1.03~17.03)	⊕⊕○○ Low
	Number of necrotic spermatozoa	2	RCTs	Not serious	Serious	Not serious	Serious	Not assessable[a]	20	20	-	MD -38.23(-141.74~65.24)	⊕⊕○○ Low
	Sperm density	3	RCTs	Not serious	Serious	Not serious	Serious	Not assessable[a]	38	40	-	MD -72.86(-96.36~-49.36)	⊕⊕○○ Low
	Sperm density	1	RCTs	Not serious	Not serious	Not serious	Not serious	Not assessable[a]	10	10	-	MD -98.00 (-115.19~-80.81)	⊕⊕⊕⊕ High
	Sperm density	2	RCTs	Not serious	Serious	Not serious	Serious	Not assessable[a]	20	20	-	MD -68.88 (-106.73~-31.03)	⊕⊕○○ Low
	Sperm density	1	RCTs	Not serious	Not serious	Not serious	Serious	Not assessable[a]	8	10	-	MD -54.00 (-72.66~-35.34)	⊕⊕⊕○ Moderate

AMH, anti-Müllerian hormone; CI, confidence interval; DHEA-S, dehydroepiandrosterone sulfate; FAI, free androgen index; FSH, follicle-stimulating hormone; LH, luteinizing hormone; MD, mean difference; OR, odds ratio; RCT, randomized controlled trial; RD, risk difference; RR, risk ratio; SHBG, sex hormone-binding globulin; SMD, standardized mean difference.
GRADE certainty symbols: ⊕⊕⊕⊕, high certainty; ⊕⊕⊕○, moderate certainty; ⊕⊕○○, low certainty; ⊕○○○, very low certainty.
a. Not assessable because fewer than 10 primary studies contributed to the outcome.

Women diagnosed with PCOS								
**Author, year**	**Interventioin**	**Comparison**	**Outcome**	**Study Type**	**Effect model**	**SMD with 95% CI**	**I^2 (%)**	**Publication bias**
Yu 2020	EI	NEI	Testosterone	RCT	Fixed effects	-0 05 (-0.11~-0.01)	0	NR
Dos Santos 2020	EI	NEI	Testosterone	RCT	Random effect	0.06 (0.28~0.16)	74	NR
Yu 2020	EI	NEI	SHBG	RCT	Fixed effects	2.11 (0.63~3.59)	8	NR
Yu 2020	EI	NEI	DHEA-S	RCT	Fixed effects	-0.07 (-0.27~-0.13)	0	NR
**Author, year**	**Interventioin**	**Comparison**	**Outcome**	**Study Type**	**Effect model**	**MD with 95% CI**	**I^2 (%)**	**Publication bias**
Yu 2020	EI	NEI	Estradiol	RCT	Fixed effects	-0.09 (-1.15~0.96)	0	NR
Yu 2020	EI	NEI	FSH	RCT	Fixed effects	-0.09 (-0.57~0.4)	0	NR
Dos Santos 2020	EI	NEI	FSH	RCT	Random effect	0.11 (-0.21~0.43)	74	NR
Yu 2020	EI	NEI	LH	RCT	Fixed effects	-0.09 (-1.15~0.96)	0	NR
Dos Santos 2020	EI	NEI	LH	RCT	Random effect	0.04 (-0.27~0.35)	74	NR
Dos Santos 2020	EI	NEI	FAI	RCT	Random effect	0.21 (0.71~1.14)	74	NR
Kazeminia 2022	EI	UCI	AMH	RCT	Random effect	0.66 (0.06~-1.26)	80.78	0.949
**Women with obesity**								
**Author, year**	**Interventioin**	**Comparison**	**Outcome**	**Study Type**	**Effect model**	**SMD with 95% CI**	**I^2 (%)**	**Publication bias**
Ruiz-Gonzalez 2024	EI	SC,NI	Testosterone	RCT	Random effect	-0.93 (-1.68~-0.18)	95.3	NR
Ruiz-Gonzalez 2024	EI	SC,NI	Testosterone	RCT	Random effect	-0.63 (-1.89~0.62)	79.4	NR
Ruiz-Gonzalez 2024	EI	SC,NI	SHBG	RCT	Random effect	0.59 (-0.21~1.39)	94	NR
Ruiz-Gonzalez 2024	EI	SC,NI	SHBG	RCT	Random effect	4.66 (3.36~5.96)	98.9	NR
Ruiz-Gonzalez 2024	EI	SC,NI	FAI	RCT	Random effect	0.24 (-0.58~1.06)	96.4	NR
Ruiz-Gonzalez 2024	EI	SC,NI	FAI	RCT	Random effect	-0.87 (-2.51~0.78)	38.6	NR
Ruiz-Gonzalez 2024	EI	SC,NI	Androstenedione	RCT	Random effect	0.05 (-1.3~1.41)	0	NR
Ruiz-Gonzalez 2024	EI	SC,NI	Androstenedione	RCT	Random effect	-0.19 (-2.1~1.71)	53.5	NR
Ruiz-Gonzalez 2024	EI	SC,NI	DHEA-S	RCT	Random effect	-1.52 (-3~-0.03)	71.2	NR
Ruiz-Gonzalez 2024	EI	SC,NI	DHEA-S	RCT	Random effect	-3.04 (-5.53~-0.54)	99.6	NR
**Author, year**	**Interventioin**	**Comparison**	**Outcome**	**Study Type**	**Effect model**	**MD with 95% CI**	**I^2 (%)**	**Publication bias**
Ruiz-Gonzalez 2024	EI	SC,NI	Estradiol	RCT	Random effect	-1.05 (-2.61~0.51)	91.3	NR
Ruiz-Gonzalez 2024	EI	SC,NI	Estradiol	RCT	Random effect	-2.36 (-5.25~0.52)	99.3	NR
Ruiz-Gonzalez 2024	EI	SC,NI	Progesterone	RCT	Random effect	-0.23 (-0.75~0.29)	61.1	NR
Ruiz-Gonzalez 2024	EI	SC,NI	FSH	RCT	Random effect	0.18 (-0.24~0.6)	97.5	NR
Ruiz-Gonzalez 2024	EI	SC,NI	LH	RCT	Random effect	0.17 (-0.97~1.3)	89.4	NR
Ruiz-Gonzalez 2024	EI	SC,NI	LH	RCT	Random effect	0.1 (-1.9~2.09)	0	NR

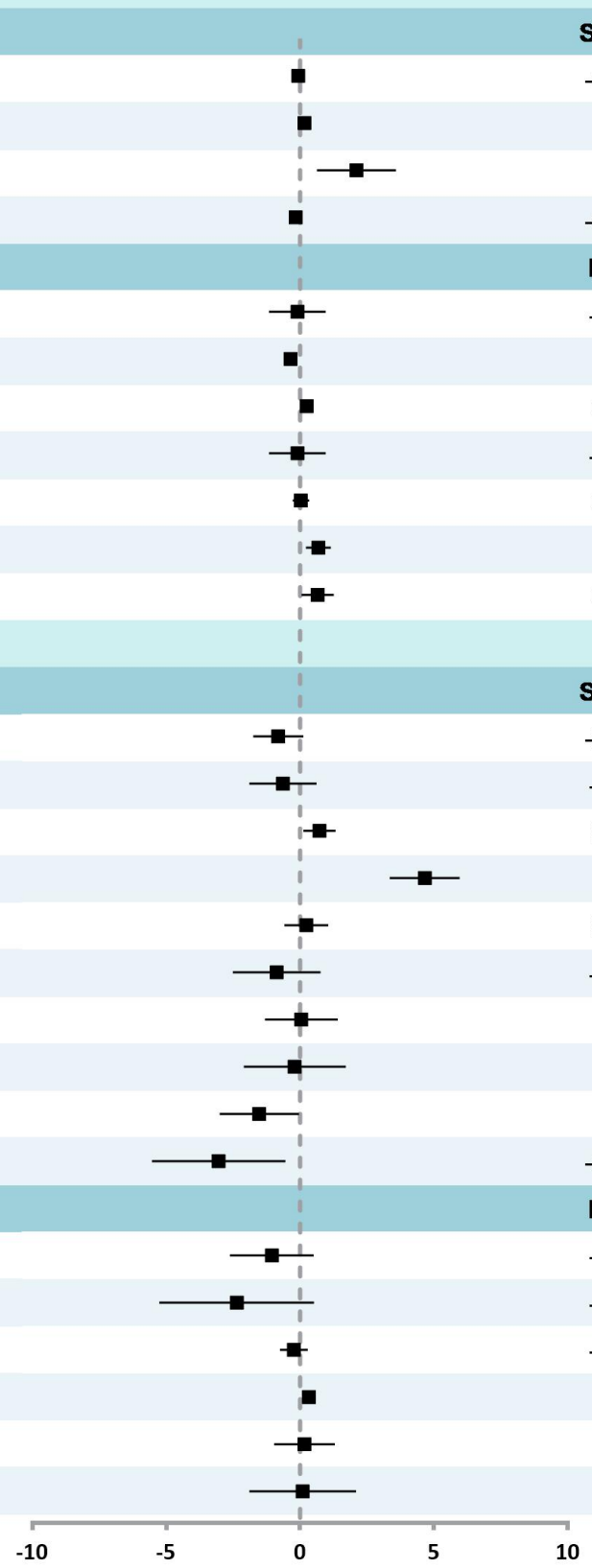

Figure S1. Narrative sensitivity analysis of exercise interventions on female hormonal outcomes stratified by population. This figure summarizes the effects of exercise interventions on hormonal parameters across different female populations. Effect sizes are presented with corresponding 95% confidence intervals, and I^2 values indicate heterogeneity. NR denotes data not reported. SMD, standardized mean difference; MD, mean difference; OR, odds ratio; RR, risk ratio; RD, risk difference; EI, exercise intervention; PA, physical activity; OA, outdoor aerobics; IA, indoor aerobics; MI, minimal intervention; NI, no intervention; NEI, non-exercise intervention; SC, standard care; PI, pharmacological intervention; DI, dietary intervention; BI, behavioral intervention; IVF, in vitro fertilization; UCI, undefined control intervention; AMH, anti-Müllerian hormone; DHEA-S, dehydroepiandrosterone sulfate; FAI, free androgen index; FSH, follicle-stimulating hormone; LH, luteinizing hormone; SHBG, sex hormone-binding globulin; RCT, randomized controlled trial. *Log-transformed.

Author, year	Interventioin	Comparison	Outcome	Study Type	Effect model	SMD with 95% CI	I^2 (%)	Publication bias
**Exercise-only interventions**								
Yu 2020	EI	NEI	Testosterone	RCT	Fixed effects	-0 05 (-0.11~-0.01)	0	NR
Dos Santos 2020	EI	NEI	Testosterone	RCT	Random effect	0.06 (0.28~0.16)	74	NR
Yu 2020	EI	NEI	SHBG	RCT	Fixed effects	2.11 (0.63~3.59)	8	NR
Yu 2020	EI	NEI	DHEA-S	RCT	Fixed effects	-0.07 (-0.27~-0.13)	0	NR
**Author, year**	**Interventioin**	**Comparison**	**Outcome**	**Study Type**	**Effect model**	**MD with 95% CI**	**I^2 (%)**	**Publication bias**
Yu 2020	EI	NEI	Estradiol	RCT	Fixed effects	-0.09 (-1.15~0.96)	0	NR
Yu 2020	EI	NEI	FSH	RCT	Fixed effects	-0.09 (-0.57~0.4)	0	NR
Dos Santos 2020	EI	NEI	FSH	RCT	Random effect	0.11 (-0.21~0.43)	74	NR
Yu 2020	EI	NEI	LH	RCT	Fixed effects	-0.09 (-1.15~0.96)	0	NR
Dos Santos 2020	EI	NEI	LH	RCT	Random effect	0.04 (-0.27~0.35)	74	NR
Dos Santos 2020	EI	NEI	FAI	RCT	Random effect	0.21 (0.71~1.14)	74	NR
Kazeminia 2022	EI	UCI	AMH	RCT	Random effect	0.66 (0.06~-1.26)	80.78	0.949
**Exercise combined with co-interventions**								
**Author, year**	**Interventioin**	**Comparison**	**Outcome**	**Study Type**	**Effect model**	**SMD with 95% CI**	**I^2 (%)**	**Publication bias**
Ruiz-Gonzalez 2024	EI	SC,NI	Testosterone	RCT	Random effect	-0.93 (-1.68~-0.18)	95.3	NR
Ruiz-Gonzalez 2024	EI	SC,NI	Testosterone	RCT	Random effect	-0.63 (-1.89~0.62)	79.4	NR
Ruiz-Gonzalez 2024	EI	SC,NI	SHBG	RCT	Random effect	0.59 (-0.21~1.39)	94	NR
Ruiz-Gonzalez 2024	EI	SC,NI	SHBG	RCT	Random effect	4.66 (3.36~5.96)	98.9	NR
Ruiz-Gonzalez 2024	EI	SC,NI	FAI	RCT	Random effect	0.24 (-0.58~1.06)	96.4	NR
Ruiz-Gonzalez 2024	EI	SC,NI	FAI	RCT	Random effect	-0.87 (-2.51~0.78)	38.6	NR
Ruiz-Gonzalez 2024	EI	SC,NI	Androstenedione	RCT	Random effect	0.05 (-1.3~1.41)	0	NR
Ruiz-Gonzalez 2024	EI	SC,NI	Androstenedione	RCT	Random effect	-0.19 (-2.1~1.71)	53.5	NR
Ruiz-Gonzalez 2024	EI	SC,NI	DHEA-S	RCT	Random effect	-1.52 (-3~-0.03)	71.2	NR
Ruiz-Gonzalez 2024	EI	SC,NI	DHEA-S	RCT	Random effect	-3.04 (-5.53~-0.54)	99.6	NR
**Author, year**	**Interventioin**	**Comparison**	**Outcome**	**Study Type**	**Effect model**	**MD with 95% CI**	**I^2 (%)**	**Publication bias**
Sustarsic 2023	PA	UCI	Testosterone	RCT	Random effect	0.12 (0.02~0.23)	0	NR
Sustarsic 2023	PA	UCI	SHBG	RCT	Random effect	5.55 (1.89~-9.21)	47	NR
Sustarsic 2023	PA	UCI	FAI	RCT	Random effect	-0.53 (-1.91~0.86)	0	NR
Ruiz-Gonzalez 2024	EI	SC,NI	Estradiol	RCT	Random effect	-1.05 (-2.61~0.51)	91.3	NR
Ruiz-Gonzalez 2024	EI	SC,NI	Estradiol	RCT	Random effect	-2.36 (-5.25~0.52)	99.3	NR
Ruiz-Gonzalez 2024	EI	SC,NI	Progesterone	RCT	Random effect	-0.23 (-0.75~0.29)	61.1	NR
Ruiz-Gonzalez 2024	EI	SC,NI	FSH	RCT	Random effect	0.18 (-0.24~0.6)	97.5	NR
Ruiz-Gonzalez 2024	EI	SC,NI	LH	RCT	Random effect	0.17 (-0.97~1.3)	89.4	NR
Ruiz-Gonzalez 2024	EI	SC,NI	LH	RCT	Random effect	0.1 (-1.9~2.09)	0	NR

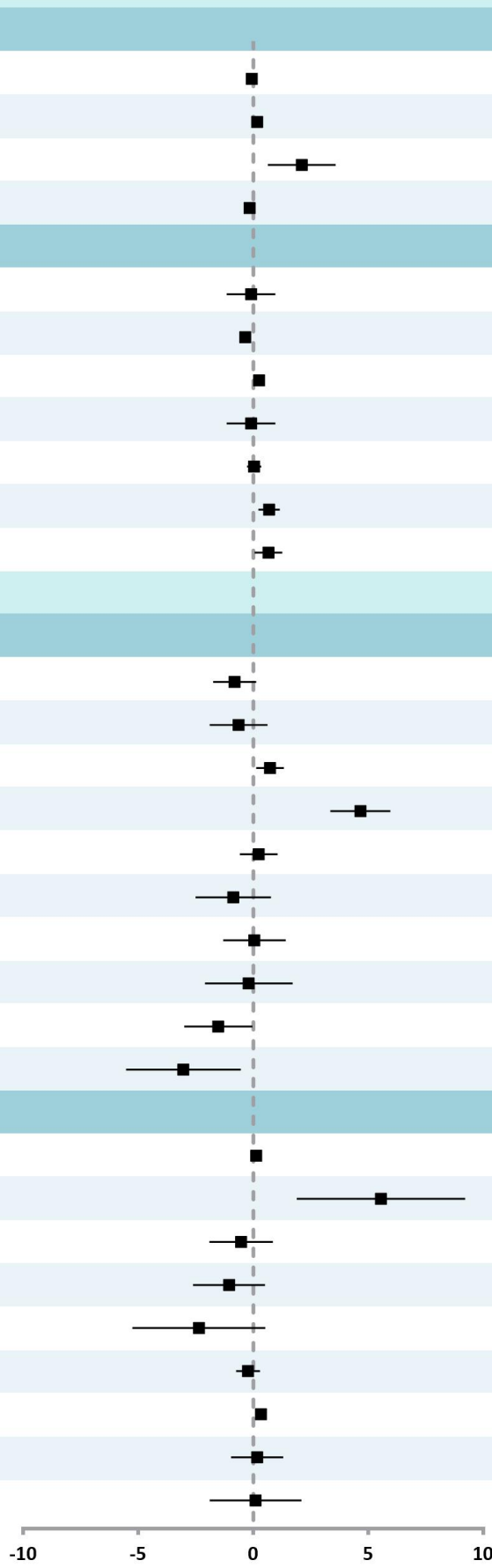

Figure S2. Narrative sensitivity analysis of exercise interventions on female hormonal outcomes stratified by intervention type. This figure summarizes hormonal outcomes according to exercise-only interventions and exercise combined with co-interventions. Effect sizes are shown with corresponding 95% confidence intervals, and I^2 values indicate heterogeneity. NR denotes data not reported. SMD, standardized mean difference; MD, mean difference; EI, exercise intervention; PA, physical activity; NEI, non-exercise intervention; SC, standard care; NI, no intervention; UCI, undefined control intervention; AMH, anti-Müllerian hormone; DHEA-S, dehydroepiandrosterone sulfate; FAI, free androgen index; FSH, follicle-stimulating hormone; LH, luteinizing hormone; SHBG, sex hormone-binding globulin; RCT, randomized controlled trial.

Exercise-only interventions

Author, year	Interventioin	Comparison	Outcome	Study Type	Effect model	OR*/RR*/RD with 95% CI	I^2 (%)	Publication bias
Hajizadeh Maleki 2022	HICT	NEI	Female Partner's Pregnancy Rate	RCT	Random effect	1.71 (0.86~2.56)	NR	0.17
Hajizadeh Maleki 2022	CET	NEI	Female Partner's Pregnancy Rate	RCT	Random effect	3.32 (2.59~4.06)	NR	0.17
Hajizadeh Maleki 2022	HIIT	NEI	Female Partner's Pregnancy Rate	RCT	Random effect	1.53 (0.66~2.40)	NR	0.17
Hajizadeh Maleki 2022	MICT	NEI	Female Partner's Pregnancy Rate	RCT	Random effect	3.28 (2.41~4.15)	NR	0.17
Hajizadeh Maleki 2022	RT	NEI	Female Partner's Pregnancy Rate	RCT	Random effect	2.53 (1.72~3.34)	NR	0.17
Lo Giudice 2024	PA	UCI	Female Partner's Pregnancy Rate	RCT	Fixed effect	0.5 (0.46~0.53)	99	NR
**Author, year**	**Interventioin**	**Comparison**	**Outcome**	**Study Type**	**Effect model**	**SMD with 95% CI**	**I^2 (%)**	**Publication bias**
Hajizadeh Maleki 2022	CET	NEI	Semen Volume	RCT	Random effect	0.49 (0.30~0.68)	0	0.16
Hajizadeh Maleki 2022	HICT	NEI	Semen Volume	RCT	Random effect	0.08 (-0.08~0.32)	0	0.16
Hajizadeh Maleki 2022	HIIT	NEI	Semen Volume	RCT	Random effect	-0.10 (-0.27~0.07)	0	0.16
Hajizadeh Maleki 2022	MICT	NEI	Semen Volume	RCT	Random effect	0.41 (0.27~0.54)	0	0.16
Hajizadeh Maleki 2022	RT	NEI	Semen volume	RCT	Random effect	-0.003 (-0.18~0.18)	0	0.16
Hajizadeh Maleki 2022	CET	NEI	Sperm Count	RCT	Random effect	46.24 (30.09 to 62.40)	8.8	0.14
Hajizadeh Maleki 2022	HIC	NEI	Sperm Count	RCT	Random effect	24.31 (8.49 to 40.14)	8.8	0.14
Hajizadeh Maleki 2022	HIIT	NEI	Sperm Count	RCT	Random effect	2.94 (-12.98 to 18.86)	8.8	0.14
Hajizadeh Maleki 2022	MICT	NEI	Sperm Count	RCT	Random effect	39.11 (25.65 to 52.55)	8.8	0.14
Hajizadeh Maleki 2022	RT	NEI	Sperm Count	RCT	Random effect	8.04 (-8.03 to 24.12)	8.8	0.14
Lo Giudice 2024	PA	UCI	Sperm Count	RCT	Fixed effect	0.63 (0.41~0.85)	60	NR
Lo Giudice 2024	PA	UCI	Sperm Total Motility	RCT	Fixed effect	0.63 (0.41~0.85)	60	NR
Hajizadeh Maleki 2022	CET	NEI	Progressive Motility	RCT	Random effect	11.54 (9.73~13.35)	0	0.08
Hajizadeh Maleki 2022	HIC	NEI	Progressive Motility	RCT	Random effect	6.02 (3.88~8.15)	0	0.08
Hajizadeh Maleki 2022	HIIT	NEI	Progressive Motility	RCT	Random effect	5.45 (3.58~7.33)	0	0.08
Hajizadeh Maleki 2022	MICT	NEI	Progressive Motility	RCT	Random effect	9.92 (7.84~12.01)	0	0.08
Hajizadeh Maleki 2022	RT	NEI	Progressive Motility	RCT	Random effect	4.12 (2.16~6.08)	0	0.08
Hajizadeh Maleki 2022	CET	NEI	Sperm Morphology	RCT	Random effect	3.33 (2.93~3.72)	0	0.62
Hajizadeh Maleki 2022	HIC	NEI	Sperm Morphology	RCT	Random effect	2.12 (1.72~2.53)	0	0.62
Hajizadeh Maleki 2022	HIIT	NEI	Sperm Morphology	RCT	Random effect	1.85 (1.43~2.27)	0	0.62
Hajizadeh Maleki 2022	MICT	NEI	Sperm Morphology	RCT	Random effect	2.68 (2.28~3.07)	0	0.62
Hajizadeh Maleki 2022	RT	NEI	Sperm Morphology	RCT	Random effect	1.82 (0.01~3.62)	0	0.62
Lo Giudice 2024	PA	UCI	Sperm Morphology	RCT	Fixed effect	0.56 (0.34~0.78)	75	NR
Hajizadeh Maleki 2022	CET	NEI	Sperm Concentration	RCT	Random effect	8.44 (6.30~10.59)	0	0.87
Hajizadeh Maleki 2022	HIC	NEI	Sperm Concentration	RCT	Random effect	2.80 (0.79~4.81)	0	0.87
Hajizadeh Maleki 2022	HIIT	NEI	Sperm Concentration	RCT	Random effect	2.27 (0.23~4.31)	0	0.87
Hajizadeh Maleki 2022	MICT	NEI	Sperm Concentration	RCT	Random effect	7.09 (5.28~8.89)	0	0.87
Hajizadeh Maleki 2022	RT	NEI	Sperm Concentration	RCT	Random effect	3.02 (0.96~5.08)	0	0.87
Lo Giudice 2024	PA	UCI	Sperm Concentration	RCT	Fixed effect	0.28 (0.05~0.52)	0	NR
Hajizadeh Maleki 2022	CET	NEI	Sperm DNA Fragmentation	RCT	Random effect	-6.20 (-10.67~-1.73)	90.7	0.09
Hajizadeh Maleki 2022	HIC	NEI	Sperm DNA Fragmentation	RCT	Random effect	-2.85 (-7.33~1.63)	90.7	0.09
Hajizadeh Maleki 2022	HIIT	NEI	Sperm DNA Fragmentation	RCT	Random effect	-2.83 (-7.33~1.67)	90.7	0.09
Hajizadeh Maleki 2022	MICT	NEI	Sperm DNA Fragmentation	RCT	Random effect	-4.33 (-7.97~-0.69)	90.7	0.09
Hajizadeh Maleki 2022	RT	NEI	Sperm DNA Fragmentation	RCT	Random effect	-3.25 (-7.76~1.24)	90.7	0.09
**Author, year**	**Interventioin**	**Comparison**	**Outcome**	**Study Type**	**Effect model**	**MD with 95% CI**	**I^2 (%)**	**Publication bias**
Song 2025	EI	NI	Sperm density	RCT	Random effect	-7.286 (-9.636~-4.936))	83	NR
Song 2025	OA	NI	Sperm density	RCT	Random effect	-9.8 (-11.519~-6.888)	NR	NR
Song 2025	IA	NI	Sperm density	RCT	Random effect	-6.888 (-1.0673~-3.103)	83	NR
Song 2025	RT	NI	Sperm density	RCT	Random effect	-5.4 (-7.266~-3.534)	NR	NR
Song 2025	EI	NI	NAS	RCT	Random effect	-1.216 (-3.952~1.519))	91	NR
Song 2025	OA	NI	NAS	RCT	Random effect	-1.843 (-6.779~3.092)	93	NR
Song 2025	IA	NI	NAS	RCT	Random effect	0.8 (-0.103~1.703)	91	NR
Song 2025	OA	NI	NNS	RCT	Random effect	-3.823 (-14.174~6.524)	95	NR

Exercise combined with co-interventions

Author, year	Interventioin	Comparison	Outcome	Study Type	Effect model	MD with 95% CI	I^2 (%)	Publication bias
Chen 2023	EI	NEI	Female Partner's Pregnancy Rate	RCT	Random effect	3.43 (2.05~4.82)	NR	NR

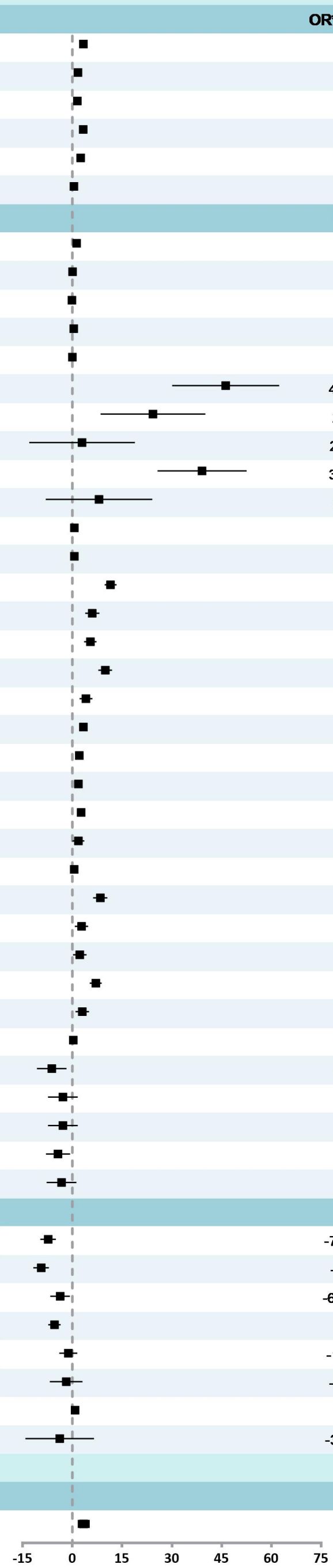

Figure S3. Narrative sensitivity analysis of exercise interventions on male fertility-related outcomes stratified by intervention type. This figure summarizes fertility-related outcomes according to exercise-only interventions and exercise combined with co-interventions. Outcomes include female partner's pregnancy rate and male semen parameters. Effect estimates are shown with corresponding 95% confidence intervals, and I^2 values indicate heterogeneity where reported. NR denotes data not reported. OR, odds ratio; RR, risk ratio; RD, risk difference; SMD, standardized mean difference; MD, mean difference; EI, exercise intervention; PA, physical activity; OA, outdoor aerobics; IA, indoor aerobics; CET, continuous exercise training; HICT, high-intensity continuous training; HIIT, high-intensity interval training; HIC, high-intensity cycling; MICT, moderate-intensity continuous training; RT, resistance training; NAS, not additionally specified; NEI, non-exercise intervention; NI, no intervention; UCI, undefined control intervention; RCT, randomized controlled trial.

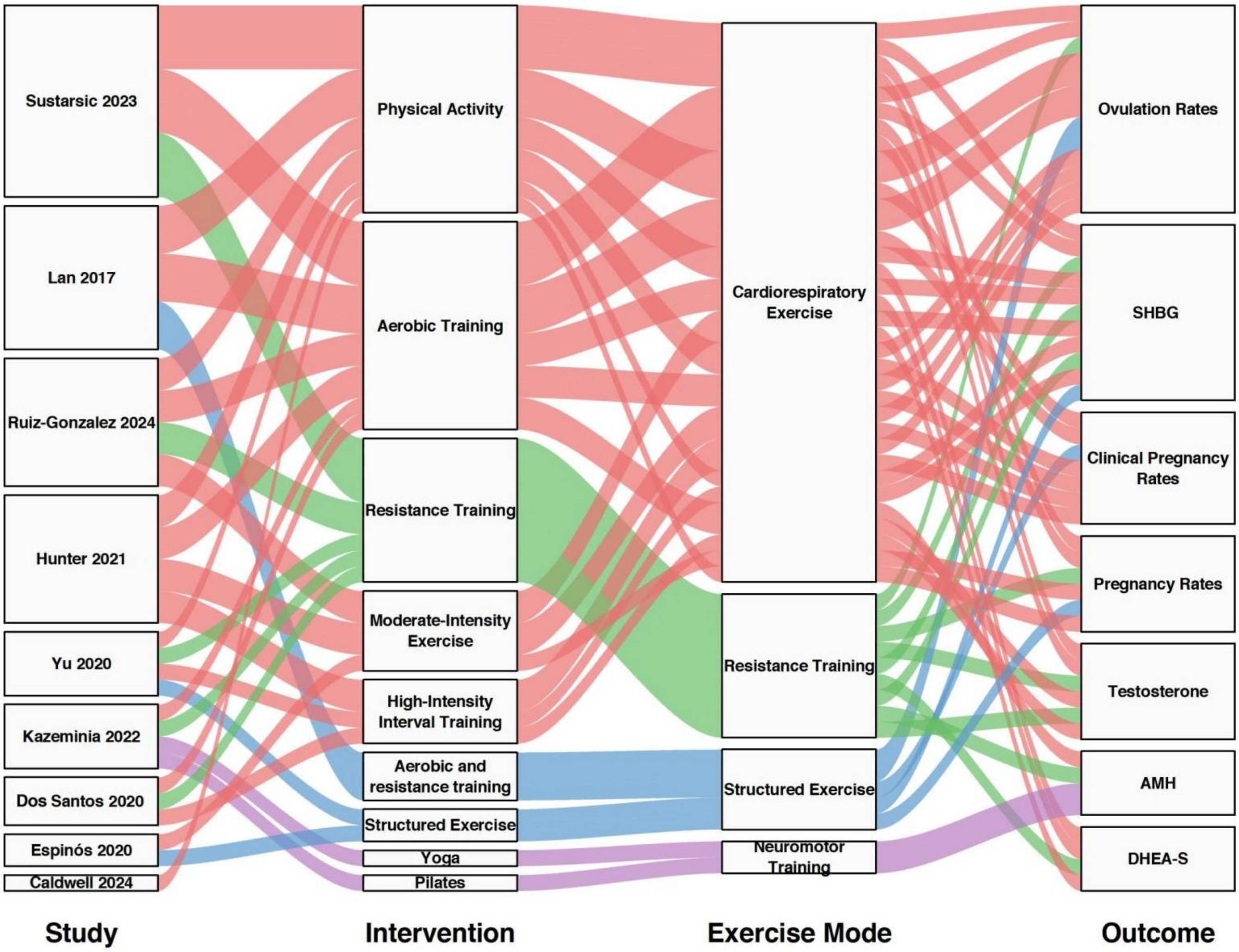

Figure S4. Alluvial diagram analysis illustrating the impacts of exercise modalities on fertility-related outcomes in females. This diagram visualizes the flow from individual studies (left) through different exercise interventions (second column) and broader exercise modes (third column) to specific fertility outcomes (right). Only studies reporting positive findings with sufficient information to map the exercise intervention, broader exercise mode, and corresponding outcome were included in this diagram. The width of each flow is proportional to the strength of the connection between study, intervention, exercise mode, and outcome, highlighting the diverse pathways through which exercise influences female reproductive health. AMH: Anti-Müllerian hormone; DHEA-S: Dehydroepiandrosterone sulfate; SHBG: Sex hormone-binding globulin.

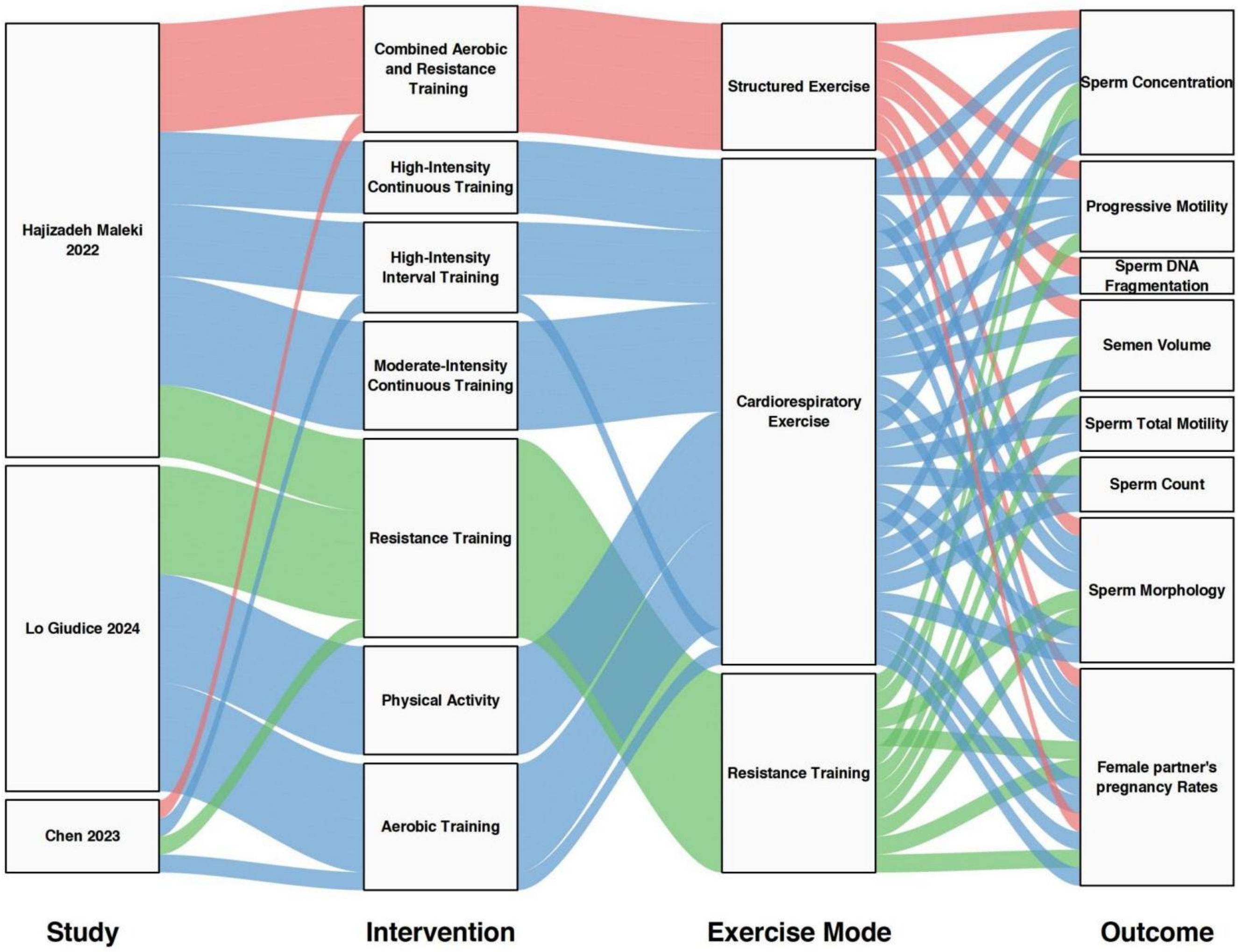

Figure S5. Alluvial diagram for the impacts of exercise modalities on fertility-related outcomes in males. This diagram visualizes the flow from individual studies (left) through different exercise interventions (second column) and broader exercise modes (third column) to specific fertility outcomes (right). Only studies reporting positive findings with sufficient information to map the exercise intervention, broader exercise mode, and corresponding outcome were included in this diagram. The width of each flow is proportional to the strength of the connection between study, intervention, exercise mode, and outcome, highlighting the diverse pathways through which exercise influences male reproductive health.

PRISMA 2020 Checklist

Section and Topic	Item #	Checklist item	Location where item is reported
**TITLE**			
Title	1	Identify the report as a systematic review.	1
**ABSTRACT**			
Abstract	2	See the PRISMA 2020 for Abstracts checklist.	2-3
**INTRODUCTION**			
Rationale	3	Describe the rationale for the review in the context of existing knowledge.	4-5
Objectives	4	Provide an explicit statement of the objective(s) or question(s) the review addresses.	5
**METHODS**			
Eligibility criteria	5	Specify the inclusion and exclusion criteria for the review and how studies were grouped for the syntheses.	7-9
Information sources	6	Specify all databases, registers, websites, organisations, reference lists and other sources searched or consulted to identify studies. Specify the date when each source was last searched or consulted.	6
Search strategy	7	Present the full search strategies for all databases, registers and websites, including any filters and limits used.	6
Selection process	8	Specify the methods used to decide whether a study met the inclusion criteria of the review, including how many reviewers screened each record and each report retrieved, whether they worked independently, and if applicable, details of automation tools used in the process.	9
Data collection process	9	Specify the methods used to collect data from reports, including how many reviewers collected data from each report, whether they worked independently, any processes for obtaining or confirming data from study investigators, and if applicable, details of automation tools used in the process.	10-11
Data items	10a	List and define all outcomes for which data were sought. Specify whether all results that were compatible with each outcome domain in each study were sought (e.g. for all measures, time points, analyses), and if not, the methods used to decide which results to collect.	8-10
	10b	List and define all other variables for which data were sought (e.g. participant and intervention characteristics, funding sources). Describe any assumptions made about any missing or unclear information.	-
Study risk of bias assessment	11	Specify the methods used to assess risk of bias in the included studies, including details of the tool(s) used, how many reviewers assessed each study and whether they worked independently, and if applicable, details of automation tools used in the process.	12-13
Effect measures	12	Specify for each outcome the effect measure(s) (e.g. risk ratio, mean difference) used in the synthesis or presentation of results.	14
Synthesis methods	13a	Describe the processes used to decide which studies were eligible for each synthesis (e.g. tabulating the study intervention characteristics and comparing against the planned groups for each synthesis (item #5)).	-
	13b	Describe any methods required to prepare the data for presentation or synthesis, such as handling of missing summary statistics, or data conversions.	14
	13c	Describe any methods used to tabulate or visually display results of individual studies and syntheses.	13-14
	13d	Describe any methods used to synthesize results and provide a rationale for the choice(s). If meta-analysis was performed, describe the model(s), method(s) to identify the presence and extent of statistical heterogeneity, and software package(s) used.	14
	13e	Describe any methods used to explore possible causes of heterogeneity among study results (e.g. subgroup analysis, meta-regression).	N/A
	13f	Describe any sensitivity analyses conducted to assess robustness of the synthesized results.	N/A
Reporting bias assessment	14	Describe any methods used to assess risk of bias due to missing results in a synthesis (arising from reporting biases).	N/A
Certainty assessment	15	Describe any methods used to assess certainty (or confidence) in the body of evidence for an outcome.	12-13

PRISMA 2020 Checklist

Section and Topic	Item #	Checklist item	Location where item is reported
**RESULTS**			
Study selection	16a	Describe the results of the search and selection process, from the number of records identified in the search to the number of studies included in the review, ideally using a flow diagram.	16
	16b	Cite studies that might appear to meet the inclusion criteria, but which were excluded, and explain why they were excluded.	-
Study characteristics	17	Cite each included study and present its characteristics.	16
Risk of bias in studies	18	Present assessments of risk of bias for each included study.	16
Results of individual studies	19	For all outcomes, present, for each study: (a) summary statistics for each group (where appropriate) and (b) an effect estimate and its precision (e.g. confidence/credible interval), ideally using structured tables or plots.	16-20
Results of syntheses	20a	For each synthesis, briefly summarise the characteristics and risk of bias among contributing studies.	16
	20b	Present results of all statistical syntheses conducted. If meta-analysis was done, present for each the summary estimate and its precision (e.g. confidence/credible interval) and measures of statistical heterogeneity. If comparing groups, describe the direction of the effect.	N/A
	20c	Present results of all investigations of possible causes of heterogeneity among study results.	N/A
	20d	Present results of all sensitivity analyses conducted to assess the robustness of the synthesized results.	N/A
Reporting biases	21	Present assessments of risk of bias due to missing results (arising from reporting biases) for each synthesis assessed.	N/A
Certainty of evidence	22	Present assessments of certainty (or confidence) in the body of evidence for each outcome assessed.	16
**DISCUSSION**			
Discussion	23a	Provide a general interpretation of the results in the context of other evidence.	21-24
	23b	Discuss any limitations of the evidence included in the review.	24-26
	23c	Discuss any limitations of the review processes used.	24-26
	23d	Discuss implications of the results for practice, policy, and future research.	21-24
**OTHER INFORMATION**			
Registration and protocol	24a	Provide registration information for the review, including register name and registration number, or state that the review was not registered.	6
	24b	Indicate where the review protocol can be accessed, or state that a protocol was not prepared.	6
	24c	Describe and explain any amendments to information provided at registration or in the protocol.	N/A
Support	25	Describe sources of financial or non-financial support for the review, and the role of the funders or sponsors in the review.	27
Competing interests	26	Declare any competing interests of review authors.	27
Availability of data, code and other materials	27	Report which of the following are publicly available and where they can be found: template data collection forms; data extracted from included studies; data used for all analyses; analytic code; any other materials used in the review.	27-28

From: Page MJ, McKenzie JE, Bossuyt PM, Boutron I, Hoffmann TC, Mulrow CD, et al. The PRISMA 2020 statement: an updated guideline for reporting systematic reviews. BMJ 2021;372:n71. doi: 10.1136/bmj.n71. This work is licensed under CC BY 4.0. To view a copy of this license, visit https://creativecommons.org/licenses/by/4.0/
